# A prognostic signature based on methionine metabolism-related genes for cervical cancer: integrated transcriptomic and experimental validation

**DOI:** 10.7717/peerj.21538

**Published:** 2026-07-21

**Authors:** Yong Luo, Xiao-Hui Xie, Xiao-Qin Huang, Hui-Quan Hu

**Affiliations:** 1 North Sichuan Medical College, Nanchong, Sichuan, China; 2 The First People’s Hospital of Neijiang, Neijiang, Sichuan, China; 3 Beijing Anzhen Nanchong Hospital, Capital Medical University & Nanchong Central Hospital, Nanchong, Sichuan, China

**Keywords:** Cervical cancer, Methionine metabolism, Prognostic model, Tumor microenvironment, Immune infiltration, Epigenetics, Drug sensitivity

## Abstract

**Background:**

Cervical cancer (CC) remains one of the most prevalent malignancies in the female reproductive system. Methionine metabolism (MM) plays a pivotal role in various biological processes and has been implicated in cancer progression. However, its mechanisms in CC remain unclear.

**Methods:**

Transcriptomic data from 305 patients in The Cancer Genome Atlas (TCGA) (training cohort) and 299 patients from the Gene Expression Omnibus (GEO) (GSE44001) (external validation cohort) were analyzed for differentially expressed MM-related genes (MM-RGs). Prognostic MM-RGs were identified using Cox regression, proportional hazards testing, and Least Absolute Shrinkage and Selection Operator (LASSO) regression. A risk model was constructed and validated. Functional enrichment (Gene Set Enrichment Analysis/Gene Set Variation Analysis (GSEA/GSVA)), *in-silico* immune infiltration (CIBERSORT) and drug sensitivity (oncoPredict) analyses were performed. Five genes were validated by Quantitative Reverse Transcription Polymerase Chain Reaction (qRT-PCR) on clinical samples.

**Results:**

Eight MM-RGs (MTHFD1, SMYD2, MSRB3, MTR, ENOPH1, DNMT3B, SLC38A7, PEMT) were identified as prognostic genes. A robust risk score model was developed, stratifying patients into high- and low-risk groups with significant differences in survival outcomes. Functional enrichment revealed pathways such as ECM–receptor interaction and focal adhesion. Immune analysis indicated altered infiltration of Tregs and mast cells. *In-silico* drug sensitivity analysis predicted 57 agents with significantly different IC50 values between the high- and low-risk groups (*p* < 0.05). Notably, agents such as Cediranib and BI-2536 exhibited markedly lower IC50 values in the high-risk cohort, suggesting their potential efficacy for advanced-stage treatment. qRT-PCR preliminarily indicated the overexpression of four genes in CC tissues within a small clinical cohort.

**Conclusion:**

This study establishes a novel MM-based prognostic model for CC and based on *in-silico* predictions suggests potential therapeutic targets through comprehensive transcriptomic and experimental validation.

## Introduction

Cervical cancer (CC) is one of the most common gynecologic malignancies worldwide, with over 660,000 new diagnoses and 350,000 deaths annually (Siegel et al., 2025). Despite advances in screening and human papillomavirus (HPV) vaccination, CC remains a leading cause of cancer-related mortality, particularly in low- and middle-income countries (Singh et al. 2022; Ng et al., 2023). Owing to its complex etiology, late diagnosis, and frequent treatment resistance, the identification of reliable prognostic biomarkers and therapeutic targets is urgently needed to improve patient stratification and guide personalized treatment strategies, as current clinical parameters often fail to accurately predict individual outcomes.

Methionine metabolism (MM), a core component of one-carbon metabolism, is essential for nucleotide biosynthesis, redox homeostasis, and methylation processes, primarily through the generation of S-adenosylmethionine (SAM) (Li et al., 2023; Li et al., 2022). Dysregulation of MM has been implicated in various cancers (Xu et al., 2024; Lin et al., 2025). Crucially, the metabolic state of tumor cells does not exist in isolation but is intricately linked to the tumor immune microenvironment (TIME). Recent advancements in single-cell and spatial transcriptomics have revealed that the CC microenvironment is a highly organized and heterogeneous landscape, where stromal components, such as cancer-associated fibroblasts (CAFs), play a pivotal role in modulating immune cell infiltration and therapeutic resistance (Wu et al., 2025). Emerging evidence suggests that MM is not only a metabolic byproduct but a central driver of epigenetic remodeling and immune evasion in the tumor microenvironment. Understanding the metabolic-epigenetic-immune crosstalk, particularly how MM influences these stromal-immune interactions in cervical cancer, is essential for identifying novel prognostic markers that reflect the underlying biological state of the tumor.

In this study, we integrated transcriptomic datasets from The Cancer Genome Atlas (TCGA), Genotype-Tissue Expression (GTEx), and GEO to identify MM-related genes (MM-RGs) with prognostic significance in CC. We constructed and validated a risk model based on these genes, assessed its association with the immune microenvironment and drug sensitivity, and further confirmed the expression of key genes *via* quantitative real-time PCR (qRT-PCR). Our findings provide new insights into the metabolic-epigenetic-immune crosstalk in CC and propose potential biomarkers for individualized prognosis and treatment.

## Material and methods

### Data acquisition and processing

Transcriptomic and clinical data of CC patients were downloaded from The Cancer Genome Atlas (TCGA-CESC) *via* the Genomic Data Commons (https://portal.gdc.cancer.gov/), including RNA sequencing (RNA-seq), survival information, and clinicopathological parameters. A total of 305 tumor samples and three adjacent normal tissues were included. For external normal controls, RNA-seq data of 10 normal cervical tissues were retrieved from the Genotype-Tissue Expression (GTEx) database (https://gtexportal.org/), accessed on January 23, 2024.

For external validation, the GSE44001 dataset (platform: GPL14951), comprising 299 CC tumor samples with disease-free survival (DFS) ≥ 30 days and complete clinical information, was obtained from the Gene Expression Omnibus (GEO) database (https://www.ncbi.nlm.nih.gov/geo). This dataset serves as a completely independent external cohort, utilizing a different technology platform (microarray) from the training set (RNA-seq), thereby providing a rigorous test for the model’s cross-platform generalizability.

A list of 68 MM-RGs was compiled from the Molecular Signatures Database (MSigDB) and Gene Ontology (GO) annotations (Table S1).

### Identification of differentially expressed genes

To identify genes differentially expressed between CC and normal tissues in the training set, the “DESeq2” package (v1.40.0) (Love, Huber & Anders, 2014) in R was used (—log_2_ fold change— > 0.5 and false discovery rate (FDR, adjusted *p*-value using the Benjamini–Hochberg method) < 0.05). Volcano plots and heatmaps were generated using the “ggplot2” (v3.5.1) (Gustavsson et al., 2022)and “pheatmap” (v1.0.12) (Gu & Hübschmann, 2022) packages, respectively.

### Candidate gene selection and functional enrichment

Candidate MM-RGs were defined as the intersection between DEGs and the 68 MM-RGs, identified using the “ggvenn” package (v0.1.10) (Chen & Boutros, 2011). A protein–protein interaction (PPI) network was constructed *via* STRING (https://string-db.org/) with a minimum required interaction score of 0.15 to ensure a broad yet reliable functional landscape. The network was visualized using Cytoscape software (v3.10.3). To identify central regulators, network topological features, including the degree of connectivity, were calculated using the NetworkAnalyzer plugin. Genes with high connectivity were designated as hub genes.

Gene Ontology (GO) and Kyoto Encyclopedia of Genes and Genomes (KEGG) enrichment analyses were performed using the “clusterProfiler” package (v4.8.3) (Wu et al., 2021) a Benjamini–Hochberg adjusted *p*-value (FDR) < 0.05 as the significance threshold. The top 10 enriched GO terms and KEGG pathways were visualized accordingly.

### Identification of prognostic genes

In order to determine whether there is a relationship between the candidate genes and the prognosis of CC patients, we integrated the expression data with the disease-free survival (DFS) time and status information from the training set. The “survival” (v 3.7.0, Liang et al., 2025) package was used for the univariate Cox regression analysis. The selection criteria included a *p*-value lower than 0.05 and a hazard ratio (HR) not equal to 1. The results were visualized using the “forestplot” (v 3.1.5, Maffeo et al., 2024) package.

Subsequently, the “cox.zph” function was used to conduct a proportional hazards (PH) assumption test (*p* > 0.05) on the results of the univariate Cox analysis. Then, the “glmnet”(v 4.1.8, He et al., 2024) package was utilized to perform a least absolute shrinkage and selection operator (LASSO) regression analysis on the genes obtained from the PH assumption test. The LASSO model with the smallest *λ* value was considered the optimal model, and the genes corresponding to this model were regarded as prognostic genes.

### Construction and validation of the risk score model

A prognostic risk score model was constructed using the expression levels and LASSO coefficients of selected genes, calculated as:

\begin{eqnarray*}\text{risk score}=\sum _{\mathrm{i}=1}^{\mathrm{n}}(\mathrm{coef}(\mathrm{genei})\times \mathrm{expr}(\mathrm{genei})) \end{eqnarray*}

where Coef_i represents the LASSO coefficient and Expr_i the normalized expression of gene i. Patients in the TCGA training cohort were stratified into high-risk and low-risk groups based on the optimal cut-off value determined by the “survminer” package (v3.7.0). Kaplan–Meier (KM) survival curves were generated, and the log-rank test was used to assess significance (*p* < 0.05). Time-dependent receiver operating characteristic (ROC) curves at 5, 7, 9 years were plotted using the “survivalROC” package (v1.0.3.1) (Blanche, Dartigues & Jacqmin-Gadda, 2013) to evaluate model performance. Validation was conducted in the GSE44001 cohort using the same formula and cut-off. Heatmaps were created with the “ComplexHeatmap” package (v2.16.0).

### Association with clinicopathological features

To assess the correlation between risk score and clinical variables (age, stage, Tumor/Node/Metastasis (T/N/M) classification), Wilcoxon rank-sum or Kruskal–Wallis tests were applied. Statistical significance was set at *p* < 0.05.

### Gene set enrichment and variation analysis

Gene Set Enrichment Analysis (GSEA) was conducted using “clusterProfiler” and the “c5.go.bp.v7.5.1.entrez.gmt” gene set from MSigDB. Genes were ranked by log_2_ fold change, and pathways with —normalized enrichment score (NES)— > 1, *p* < 0.05, and false discovery rate (FDR) < 0.05 were considered significant. Gene Set Variation Analysis (GSVA) was performed using the “GSVA” and “limma” packages. Single-sample GSEA (ssGSEA) scores were calculated, and pathway differences between risk groups were identified (—t— ≥ 2, *p* < 0.05). Results were visualized with “ggplot2”.

### *In-silico* immune infiltration analysis

Immune cell infiltration was estimated using the CIBERSORT algorithm (v2.1.0) with 1,000 permutations. Only samples with *p*-value < 0.05 were retained. Wilcoxon tests were used to compare immune cell proportions between groups. Correlation among immune cells and with prognostic genes was evaluated using the “psych” package (v2.4.6.26) (Ma et al., 2023) *via* Spearman correlation (—cor— > 0.3, *p* < 0.05).

### *In-silico* drug sensitivity and immunotherapy prediction

The half-maximal inhibitory concentration (IC50) values for 198 chemotherapeutic agents from the Genomics of Drug Sensitivity in Cancer (GDSC) database were estimated using the ‘oncoPredict’ R package (v1.2). To evaluate the differential therapeutic response, Wilcoxon rank-sum tests were applied to compare the predicted IC50 values between high- and low-risk groups. A total of 57 drugs exhibited significantly different IC50 values (*p* < 0.05) between the two groups. Specifically, agents with significantly lower IC50 in the high-risk group were suggested as potential candidates for advanced-stage treatment, while those with significantly lower IC50 in the low-risk group were analyzed to bridge the therapeutic gap for early-stage or low-risk patients.

Tumor Immune Dysfunction and Exclusion (TIDE) scores were calculated (http://tide.dfci.harvard.edu/) to evaluate potential response to immune checkpoint blockade. Higher TIDE scores indicate increased immune evasion and reduced immunotherapy efficacy.

### Quantitative Real-Time PCR

Five paired CC tumor and adjacent normal tissues were obtained from patients undergoing surgical resection. The clinical and histopathological characteristics of these patients, including histological type, FIGO stage, and tumor grade, are detailed in Table S2. To ensure diagnostic accuracy, representative histopathologic images of each resected tumor were blindly reviewed and described by an independent pathologist, as presented in Supplementary Document S1. This study was approved by the Ethics Review Committee of Neijiang First People’s Hospital (Approval No. 2025-ERC-036-01). All participants provided written informed consent in accordance with the Declaration of Helsinki. Total RNA was extracted using Trizol reagent, the specific sequences and melting temperatures (Tm) for all primers used in this study are listed in Table S3, and reverse transcription was performed using a cDNA synthesis kit. qRT-PCR was conducted using SYBR Green master mix on a LightCycler 480 system. Relative expression levels were calculated using the 2ˆ−ΔΔCT method. GAPDH served as the internal control. Statistical comparisons were performed using paired t-tests or Wilcoxon signed-rank tests where appropriate.

### Statistical analysis

All statistical analyses were performed using R version 4.3.3. All statistical tests were two-sided, and a *p*-value < 0.05 was considered to indicate statistical significance.

### Code availability and reproducibility

To ensure the transparency and reproducibility of the findings, the complete R analytical pipeline used in this study has been documented in a comprehensive script (code-all.R). This script includes detailed annotations for every analytical step, corresponding to each figure and table presented in the Results section. It specifies the R version (v4.3.3), all required software packages (*e.g.*, DESeq2, clusterProfiler, survival), and their respective installation instructions from CRAN, Bioconductor, or GitHub. All analyses, including CIBERSORT and Univariate Cox regression, are designed to be executed using relative paths within a structured project directory, enabling full reproduction of the findings under well-documented conditions. To ensure a streamlined workflow, we provide clear instructions for environment setup and script execution, rather than implying a universally guaranteed one-click implementation across all computational platforms. This approach ensures that researchers can accurately replicate our analysis within a compatible R environment.

### Single-cell RNA-seq data analysis

scRNA-seq data from the E-MTAB-11948 dataset, comprising three CC samples and two normal controls, were utilized for validation. The gene expression matrices (matrix.mtx, barcodes.tsv, and features.tsv) generated *via* Cell Ranger were processed using the Seurat R package (v4.3.0). Initial filtering was applied to retain genes expressed in at least three cells and cells detecting at least 200 genes.

Quality control (QC) was performed to exclude low-quality cells based on the following criteria: (1) detected gene counts (nFeature_RNA) between 200 and 6,000; (2) total RNA counts (nCount_RNA) below 30,000; and (3) a mitochondrial gene proportion (percent.mt) of less than 20%. Following QC, data were normalized using the LogNormalize method with a scale factor of 10,000. The top 2,000 highly variable genes (HVGs) were identified using the “vst” method. After scaling the data with the ScaleData function, Principal Component Analysis (PCA) was conducted to calculate the first 50 principal components.

To eliminate batch effects among samples, the Harmony algorithm was employed for integration. Cell clustering was performed based on the first 20 Harmony dimensions using the Louvain algorithm (FindNeighbors and FindClusters functions) with a resolution of 0.1, resulting in the identification of 14 cell sub-clusters. For visualization, Uniform Manifold Approximation and Projection (UMAP) was implemented using the same 20 dimensions. The metabolic activity of individual cells was subsequently quantified *via* AUCell (v1.20.2), and malignant sub-clusters were identified through inferCNV analysis using macrophages as the reference.

## Results

### Identification of MM-related differentially expressed genes

**Figure 1:**
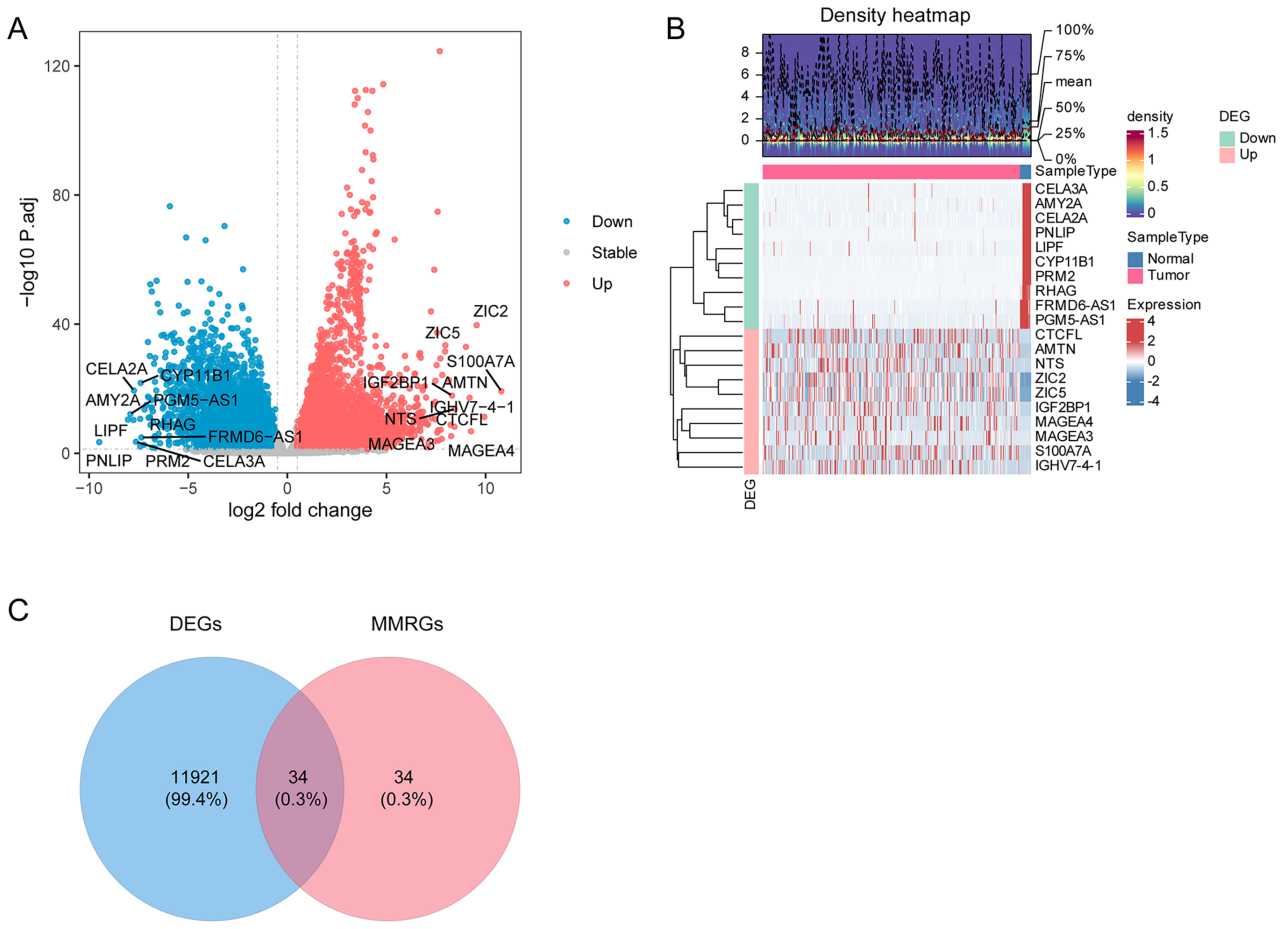
Identification of methionine metabolism-related differentially expressed genes (MM-RGs) in cervical cancer. (A) Volcano plot showing differentially expressed genes (DEGs) between cervical cancer and adjacent normal tissues (adjusted *p* < 0.05, —log_2_FC— > 0.5). Red dots represent upregulated genes, while blue dots indicate downregulated genes. (B) Heatmap of the top 20 DEGs ranked by fold change. Tumor and normal samples are clearly separated. (C) Venn diagram illustrating the overlap between DEGs (*n* = 11,955) and 68 methionine metabolism-related genes, resulting in 34 MM-RGs for further analysis.

A total of 11,955 DEGs were identified between CC (*n* = 305) and normal cervical tissues (*n* = 13; three adjacent normal and 10 from GTEx), including 6,163 upregulated and 5,792 downregulated genes (adjusted *p* < 0.05, —log_2_FC— > 0.5) (Fig. 1A). To evaluate the statistical robustness and biological relevance of these findings, we analyzed the FDR distribution of all detected genes (Fig. S1). The distribution demonstrates a significant number of genes with extremely low adjusted *p*-values (extending to 10^−^^1^^2^^4^⋅^5^^4^), indicating a profound and broad transcriptomic reprogramming in CC. While the large sample size of the TCGA cohort provides high statistical power to detect extensive variations, many of these DEGs may represent secondary effects. To minimize biological noise and focus on core metabolic drivers, we subsequently intersected these 11,955 DEGs with a curated list of 68 MM-RGs. This rigorous filtering narrowed the focus to 34 high-confidence candidate genes (representing only 0.3% of the initial DEGs), ensuring the biological specificity of the subsequent analysis. The top 20 DEGs based on fold change were visualized in a heatmap, clearly separating tumor and normal samples (Fig. 1B).

To identify MM-RGs with potential relevance in CC, we intersected the 11,955 DEGs with a curated list of 68 MM-RGs. This analysis yielded 34 overlapping genes, which were designated as candidate MM-RGs for subsequent analysis (Fig. 1C).

### Functional enrichment and protein-protein interaction network

**Figure 2:**
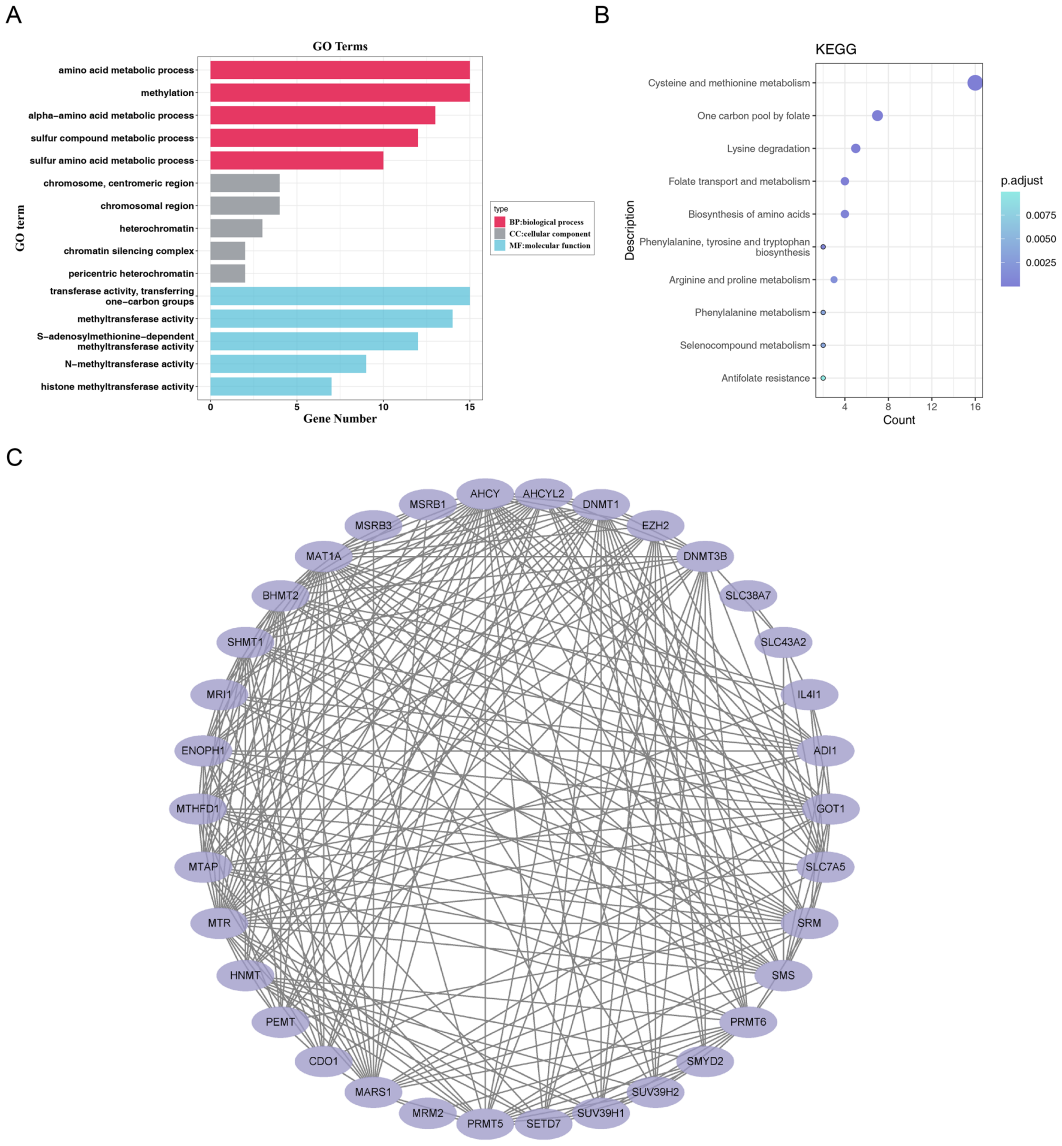
Functional enrichment of candidate MM-RGs. (A) GO enrichment analysis. (B) KEGG pathway analysis. Statistical significance was defined as FDR < 0.05. (C) PPI network of candidate MM-RGs. The functional interactome (34 nodes, 260 edges) was constructed using the STRING database (score ≥ 0.15) and visualized *via* Cytoscape. Nodes are uniformly colored in light blue to represent candidate proteins.

Enrichment analysis of 34 candidate genes revealed significant involvement in amino acid metabolic processes, methylation, and the centromeric region (Fig. 2A). Key KEGG pathways included ‘Cysteine and MM’ and ‘One-carbon pool by folate’ (Fig. 2B). The comprehensive enrichment data and associated FDR values are documented in Table S4. The PPI network (34 nodes, 260 edges) identified MTHFD1 and DNMT3B as core hub genes, suggesting a coordinated regulatory module in CC progression (Fig. 2C). The resulting network comprises 34 nodes, each representing a protein corresponding to a candidate gene, and 260 edges representing validated or predicted functional associations. In this visualization, all nodes are uniformly colored in light blue to maintain clarity and focus on the overall network topology, while gray edges indicate the presence of functional links confirmed by the STRING database. The PPI network exhibited robust connectivity, with an average node degree of 15.3. Quantitative topological analysis confirmed that all 34 nodes were interconnected with a minimum degree of ≥ 1 thereby verifying the absence of isolated nodes. This high network density suggests extensive functional synergy among these proteins. These findings indicate that the identified genes likely operate as a coordinated regulatory module, particularly in core biological processes such as amino acid metabolism and methylation, thereby contributing to the progression of CC.

### Construction of a prognostic risk model based on MM-RGs

**Figure 3:**
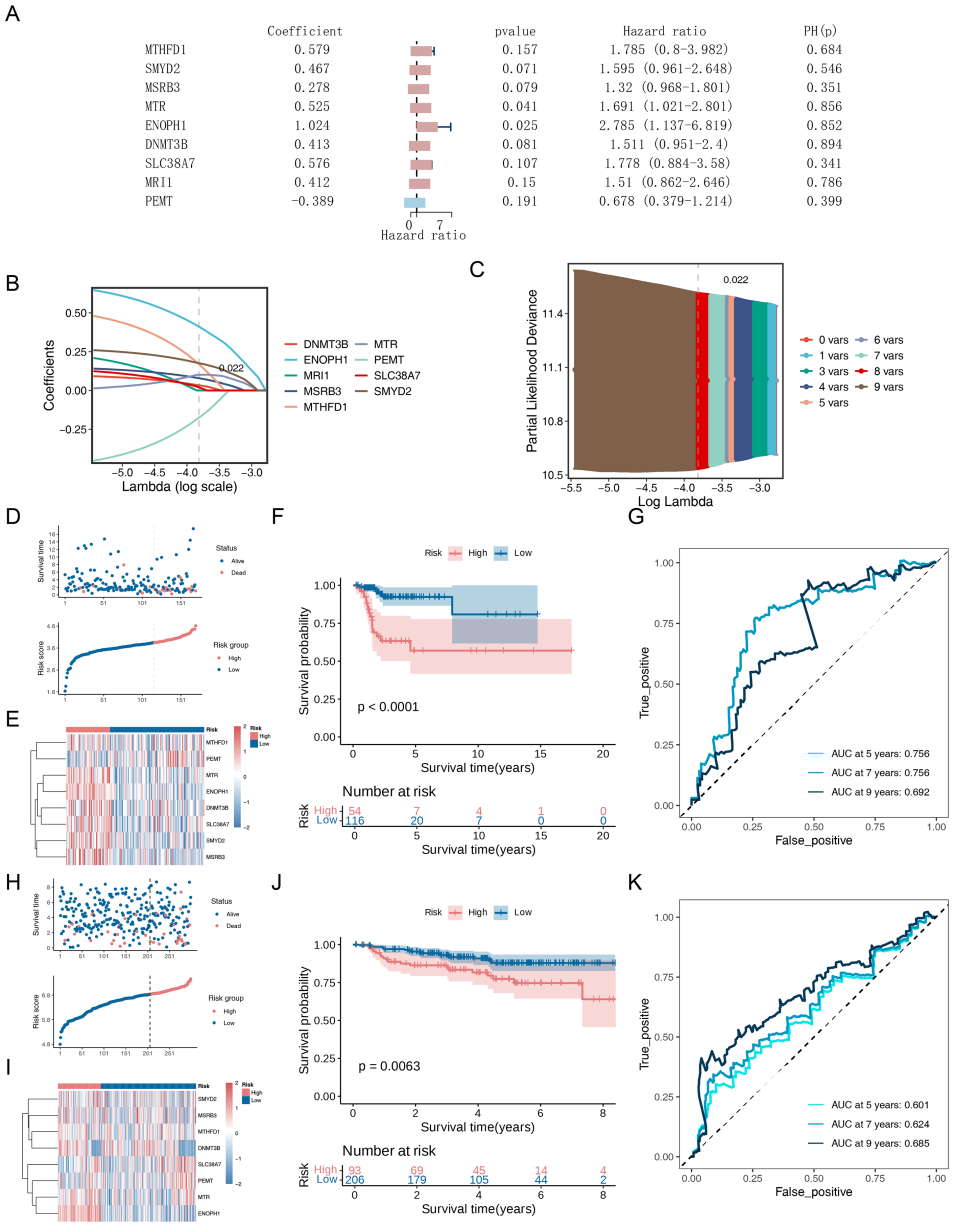
Construction and validation of the MM-RG-based prognostic model in the TCGA cohort. (A) Forest plot of univariate Cox regression analysis for the nine prognostic MM-RGs. (B) LASSO coefficient profiles of the candidate genes. (C) 10-fold cross-validation for tuning parameter selection in the LASSO model. (D) Risk score distribution and survival status of patients in the TCGA cohort. (E) Heatmap of the expression levels of the eight genes in high- and low-risk groups. (F) Kaplan–Meier survival analysis of disease-free survival (DFS) in high- and low-risk groups (log-rank *p* < 0.0001). (G) Time-dependent ROC curves evaluating the predictive performance of the model at 5, 7, and 9 years (AUC = 0.756, 0.756, and 0.692, respectively). (H) Risk score distribution and survival status in the GEO validation cohort (GSE44001). (I) Heatmap of MM-RG expression in high- and low-risk groups in the GEO cohort. (J) Kaplan–Meier survival analysis in the validation cohort (*p* = 0.0063). (K) Time-dependent ROC curves in the validation cohort (AUC = 0.601, 0.624, and 0.685 at 5, 7, and 9 years).

Univariate Cox and LASSO regression analysis identified an eight-gene signature (MTHFD1, SMYD2, MSRB3, MTR, ENOPH1, DNMT3B, SLC38A7, and PEMT) significantly associated with DFS (Figs. 3A–3D). Patients were stratified into risk groups based on the calculated risk scores. Survival analysis revealed that the high-risk group exhibited markedly poorer DFS compared to the low-risk group (log-rank *p* < 0.0001; Fig. 3E). ROC curves demonstrated robust predictive performance, with AUC values of 0.75, 0.75, and 0.69 at 5, 7, and 9 years, respectively (Fig. 3F). These results demonstrate the robust and sustained predictive accuracy of the model, particularly for long-term survival. Most signature genes were upregulated in high-risk patients, except for PEMT (Fig. 3G). These findings were successfully validated in the independent GSE44001 cohort (*n* = 299), which showed consistent survival differences and comparable AUC values (Figs. 3H–3K), confirming the robustness and cross-platform stability of the prognostic signature.

### Association between risk score and clinical features

**Figure 4:**
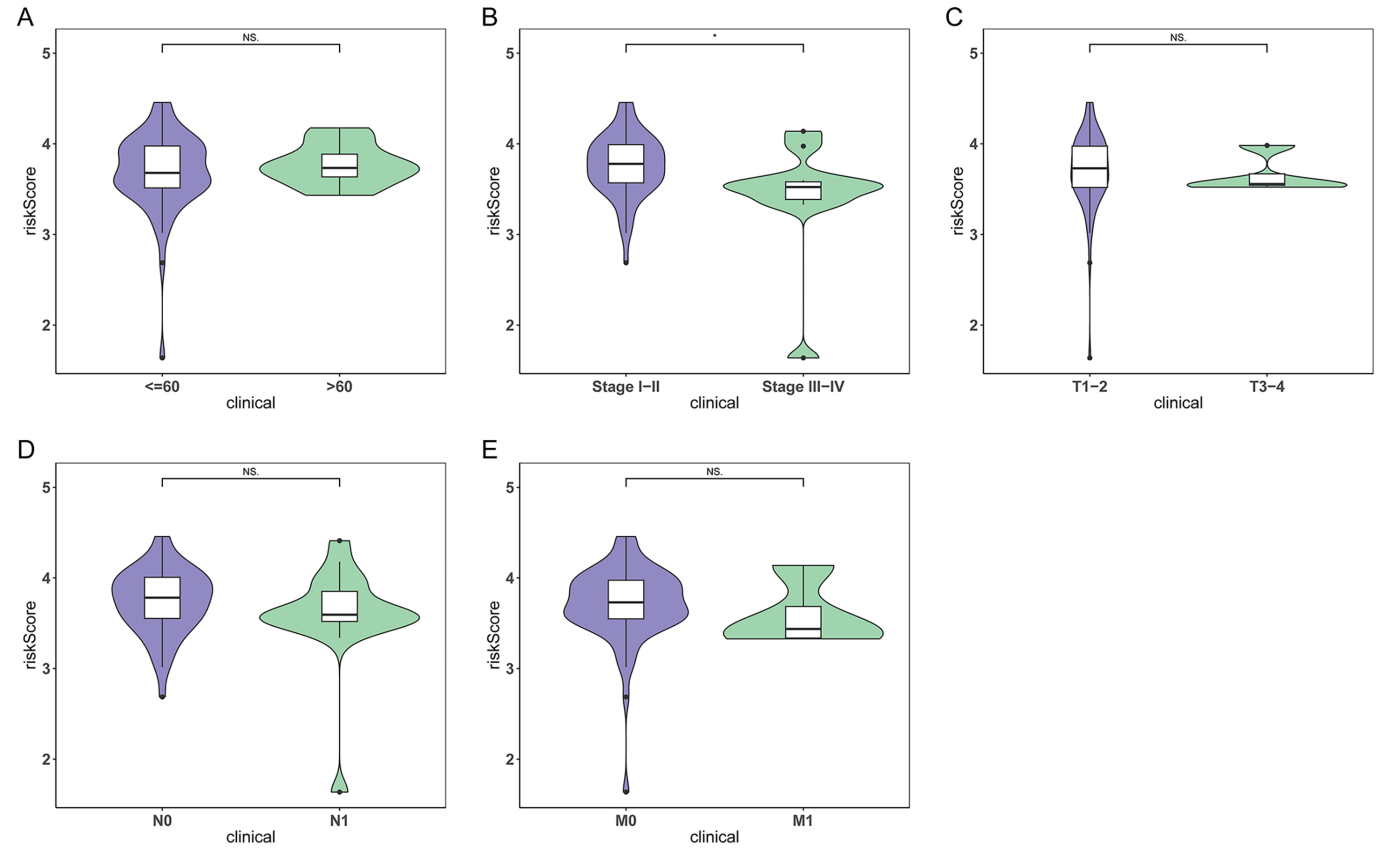
Correlation between risk score and clinicopathological characteristics. (A–E) Boxplots comparing the risk scores across different clinical subgroups including stage, age, histological grade, and lymph node status. Significant differences were observed between early and advanced stages (*p* < 0.01). The asterisk (*) indicates statistical significance at *p* < 0.05.

Analysis of clinicopathological parameters revealed that the risk score was significantly higher in advanced-stage patients (Stage III–IV *vs.* Stage I–II, *p* < 0.01), suggesting that the model is associated with disease progression (Figs. 4A–4E). No significant differences were observed in other clinical subgroups such as age or T/N stage.

### Functional characterization of high- and low-risk groups

**Figure 5:**
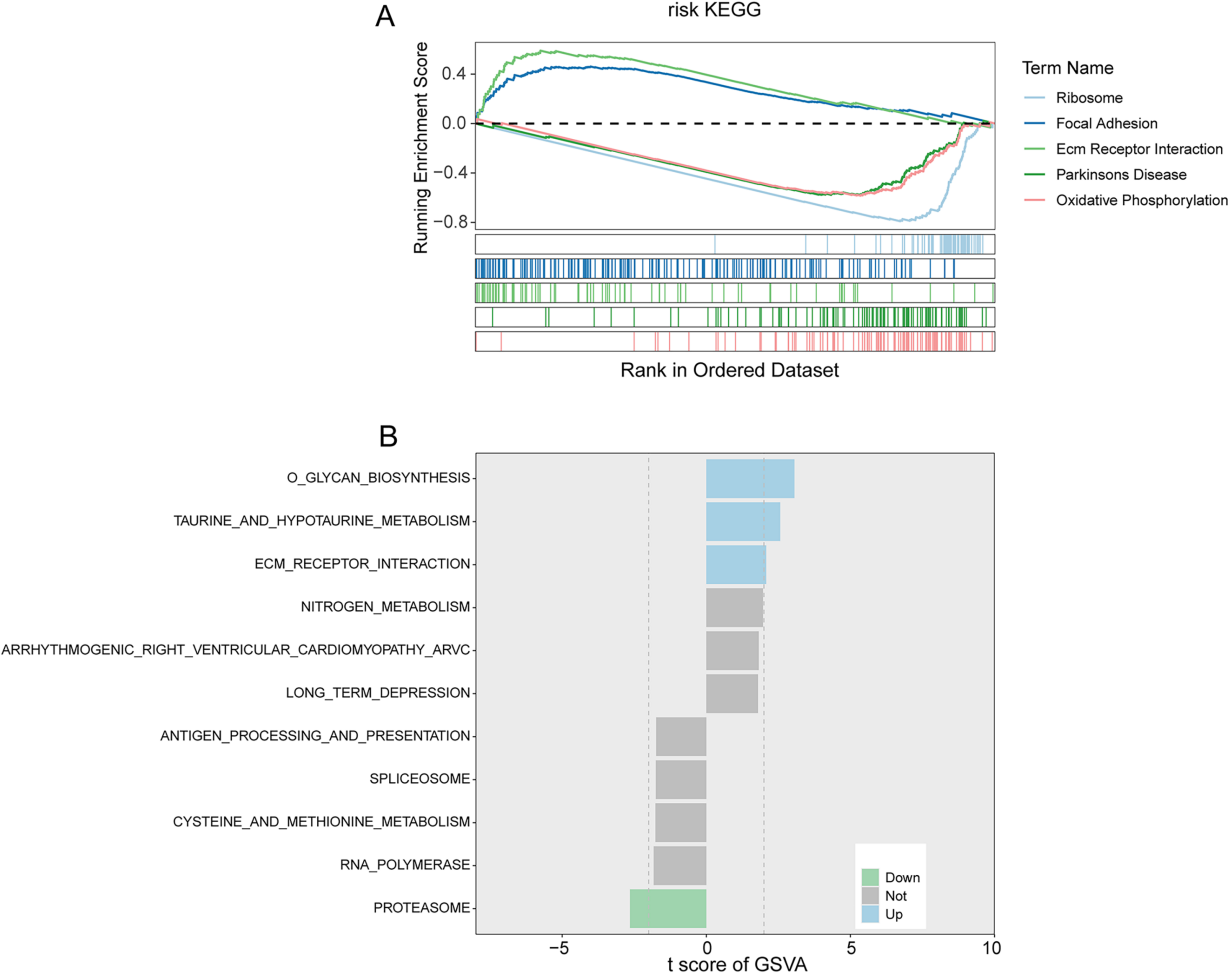
Functional enrichment analysis based on risk group stratification. (A) Gene Set Enrichment Analysis (GSEA) identifying pathways enriched in the high-risk group, including focal adhesion, ECM-receptor interaction, and oxidative phosphorylation. (B) Gene Set Variation Analysis (GSVA) showing differential pathway activity between high- and low-risk groups, with enrichment of O-glycan biosynthesis and proteasome pathways in the high-risk group.

GSEA identified 39 significantly enriched pathways between the high- and low-risk groups. The top five pathways enriched in the high-risk group were Focal Adhesion, ECM-Receptor Interaction, Parkinson’s Disease, Oxidative Phosphorylation, and Ribosome (Fig. 5A). These pathways are closely linked to tumor invasion, metabolic reprogramming, and protein synthesis.

GSVA further revealed differential pathway activity. High-risk patients showed increased enrichment of O-Glycan Biosynthesis, Taurine and Hypotaurine Metabolism, ECM-Receptor Interaction, and Proteasome pathways (Fig. 5B).

### Immune landscape between risk subgroups

**Figure 6:**
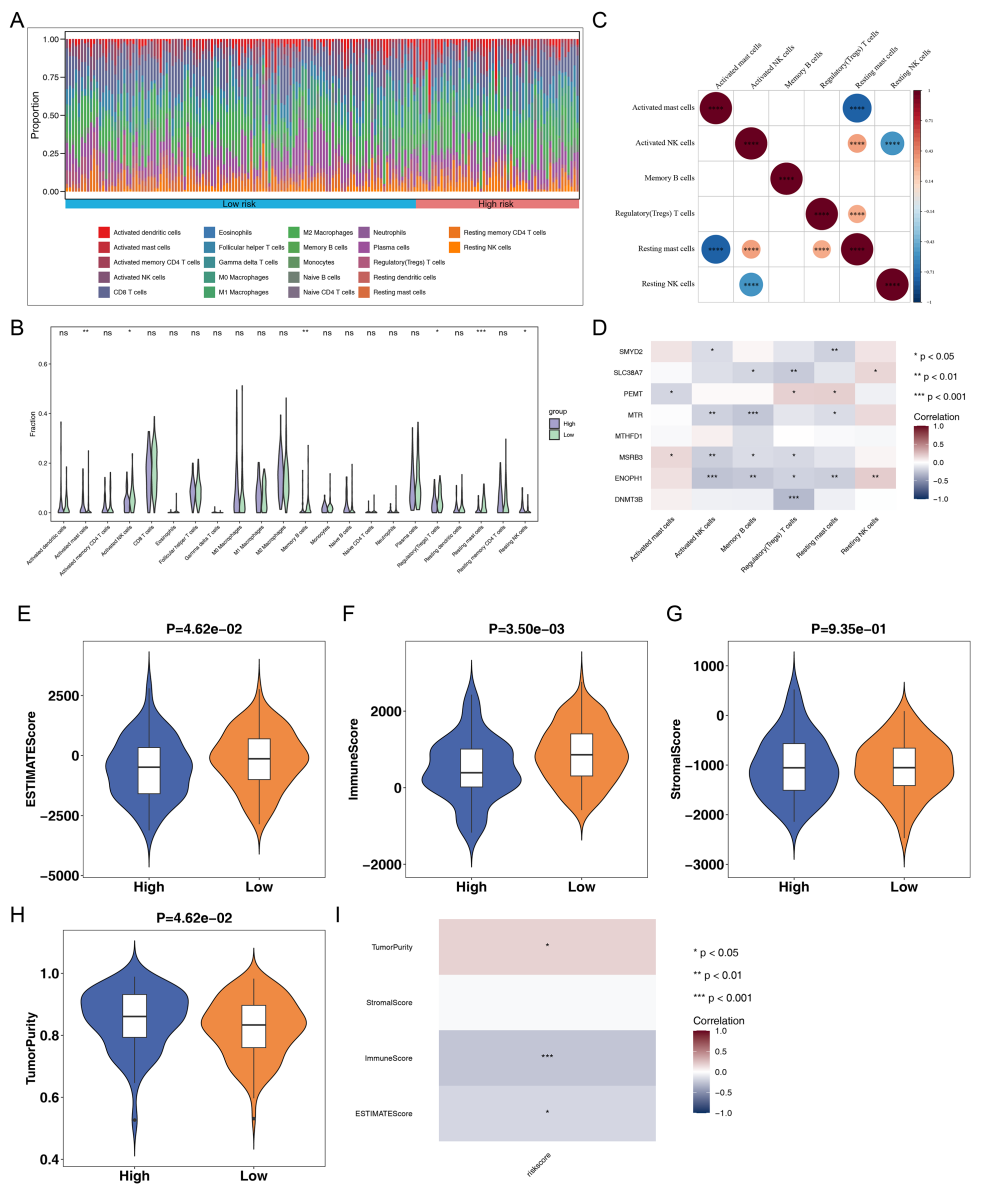
Comparison of immune infiltration between high- and low-risk groups. (A) Bar graph showing proportions of 22 immune cell types calculated by CIBERSORT. (B) Boxplots of significantly different immune cell populations between groups. (C) Correlation heatmap among significantly altered immune cells. (D) Spearman correlation between prognostic MM-RGs and immune cell types. (E–H) Comparison of tumor purity, ESTIMATE score, immune score, and stromal score between groups; significant differences observed (*p* < 0.01). (I) Expression analysis of immune checkpoint-related genes in high- *vs.* low-risk groups. The asterisks denote statistical significance as follows: * *p* < 0.05; ** *p* < 0.01; *** *p* < 0.001.

CIBERSORT analysis revealed significant differences in immune infiltration between risk groups. Specifically, six immune cell types were differentially distributed: activated mast cells, resting mast cells, activated NK cells, resting NK cells, memory B cells, and regulatory T cells (Tregs) (Figs. 6A–6B). Correlation analysis showed a strong positive correlation between Tregs and activated mast cells (Fig. 6C).

Spearman correlation analysis between prognostic genes and immune cells highlighted that MTR was positively correlated with memory B cells, while DNMT3B was negatively correlated with Tregs (Fig. 6D). Moreover, tumor purity, ESTIMATE score, and immune score differed significantly between high- and low-risk groups, further supporting the immunological relevance of the risk model (Figs. 6E–6I).

### Chemotherapy sensitivity and immunotherapy prediction

**Figure 7:**
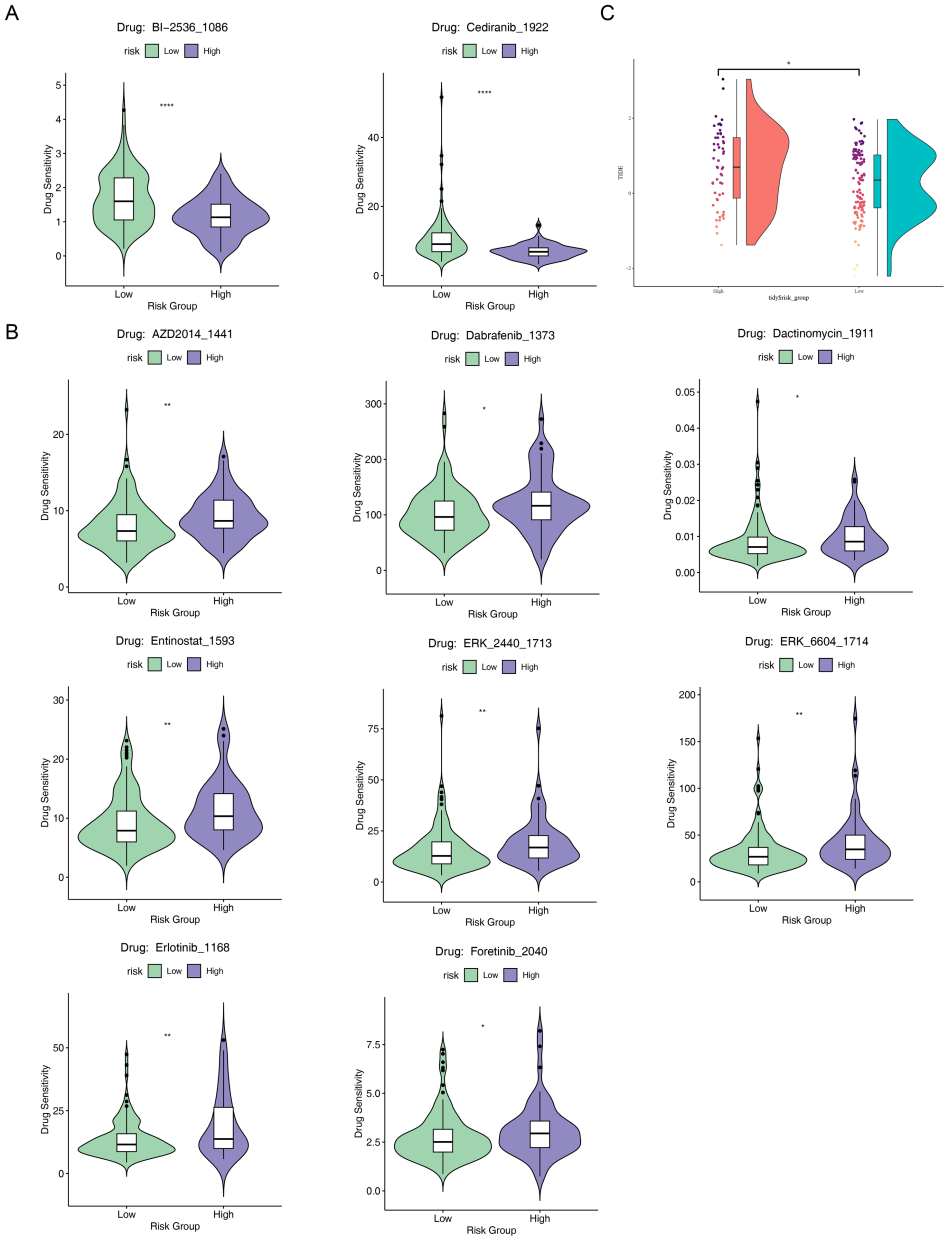
Drug sensitivity and immunotherapy response prediction. (A) Predicted IC50 values of chemotherapy agents significantly more sensitive in the high-risk group (*e.g.*, Cediranib, BI-2536). (B) Predicted IC50 values of agents showing higher sensitivity in the low-risk group (*e.g.*, AZD2014_1441, Erlotinib_1168). (C) Comparison of Tumor Immune Dysfunction and Exclusion (TIDE) scores between risk groups, indicating the potential for immunotherapy evasion. The asterisks denote statistical significance as follows: * *p* < 0.05; ** *p* < 0.01.

Predicted IC50 values for 57 chemotherapeutic drugs showed significant differences between the risk groups (all *p* < 0.05). Among these, 21 agents exhibited significantly higher sensitivity (lower IC50) in the high-risk group. Specifically, the IC50 of candidates like Cediranib, Tozasertib, and BI-2536 was substantially reduced in high-risk patients compared to the low-risk group, indicating potential therapeutic advantages. A retrospective analysis identified 21 drugs with significantly higher sensitivity (lower IC50) in the low-risk group. Key agents among these included AZD2014 (Vistusertib), Dabrafenib, Dactinomycin, Entinostat, ERK inhibitors, Erlotinib, and Foretinib (Figs. 7A–7B). These findings suggest that the metabolic risk model may potentially suggest candidate therapeutic options for high-risk patients, although these computational predictions require further experimental validation to confirm their clinical efficacy. Detailed sensitivity data for all 57 drugs are provided in Table S5. TIDE analysis revealed that the high-risk group had higher TIDE scores, indicating potential resistance to immune checkpoint blockade (Fig. 7C).

### Experimental validation by qRT-PCR

**Figure 8:**
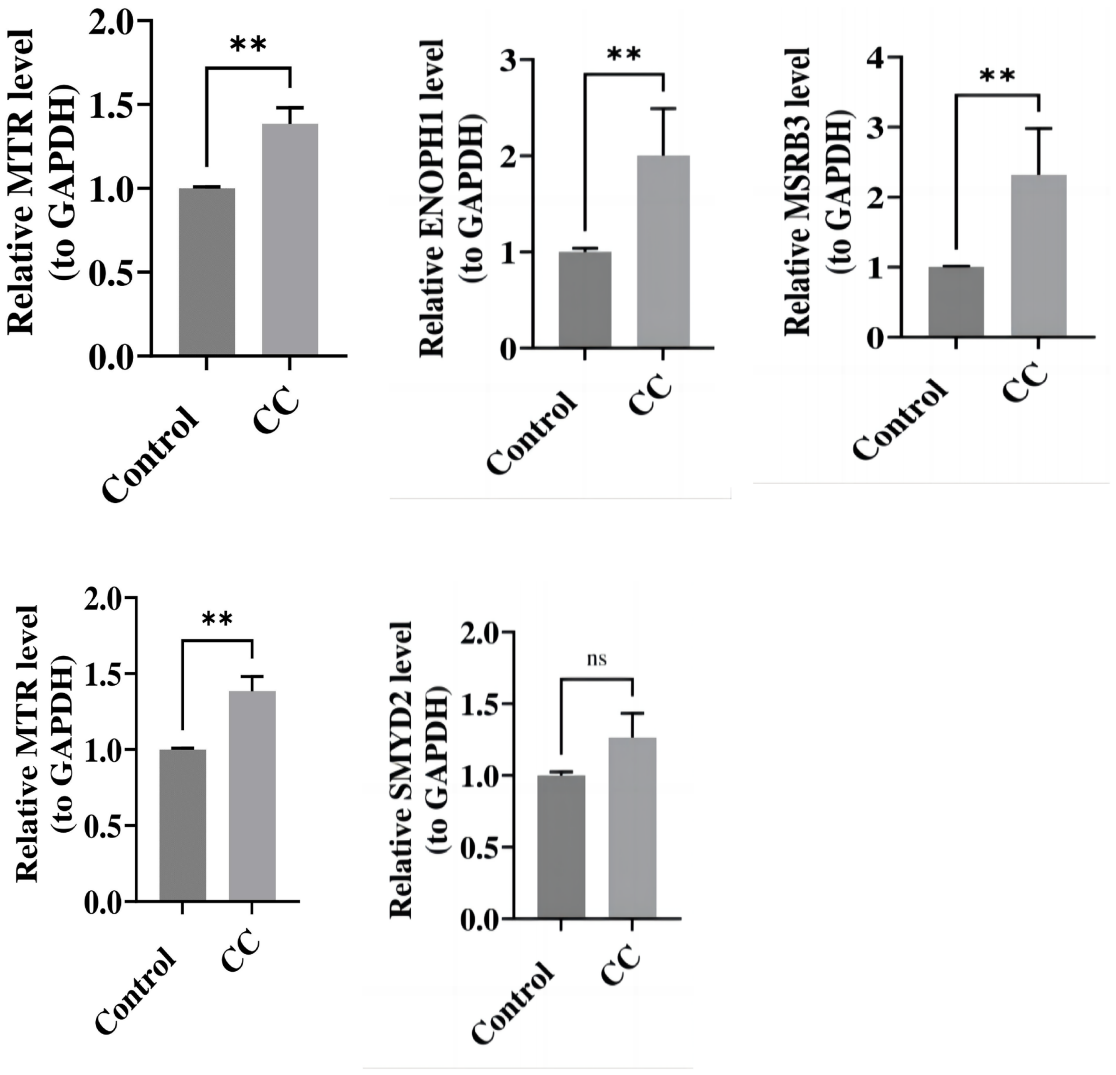
RT-qPCR confirmed that the expression trends of the five genes in CC tissues were consistent with bioinformatics predictions, with MTHFD1, ENOPH1, MTR, and MSRB3 showing significantly higher expression in tumor tissues (*P* < 0.001). The asterisks (**) indicate statistical significance at *p* < 0.01.

qRT-PCR was conducted on five paired CC tumor and adjacent normal tissues representing various clinical stages and histological subtypes to validate the expression of five key MM-RGs. The malignant nature of the tumor samples was histologically confirmed through blinded pathological evaluation (Supplementary Document S1). MTHFD1, ENOPH1, MTR, and MSRB3 were significantly upregulated in tumor tissues (*p* < 0.001), consistent with bioinformatic analysis. SMYD2 showed an upward trend but did not reach statistical significance. These findings are consistent with our bioinformatic predictions and provide preliminary evidence for the expression patterns of these genes in clinical samples, although further validation in a larger cohort is warranted (Fig. 8).

### Single-cell resolution validation of prognostic MM-RGs

**Figure 9:**
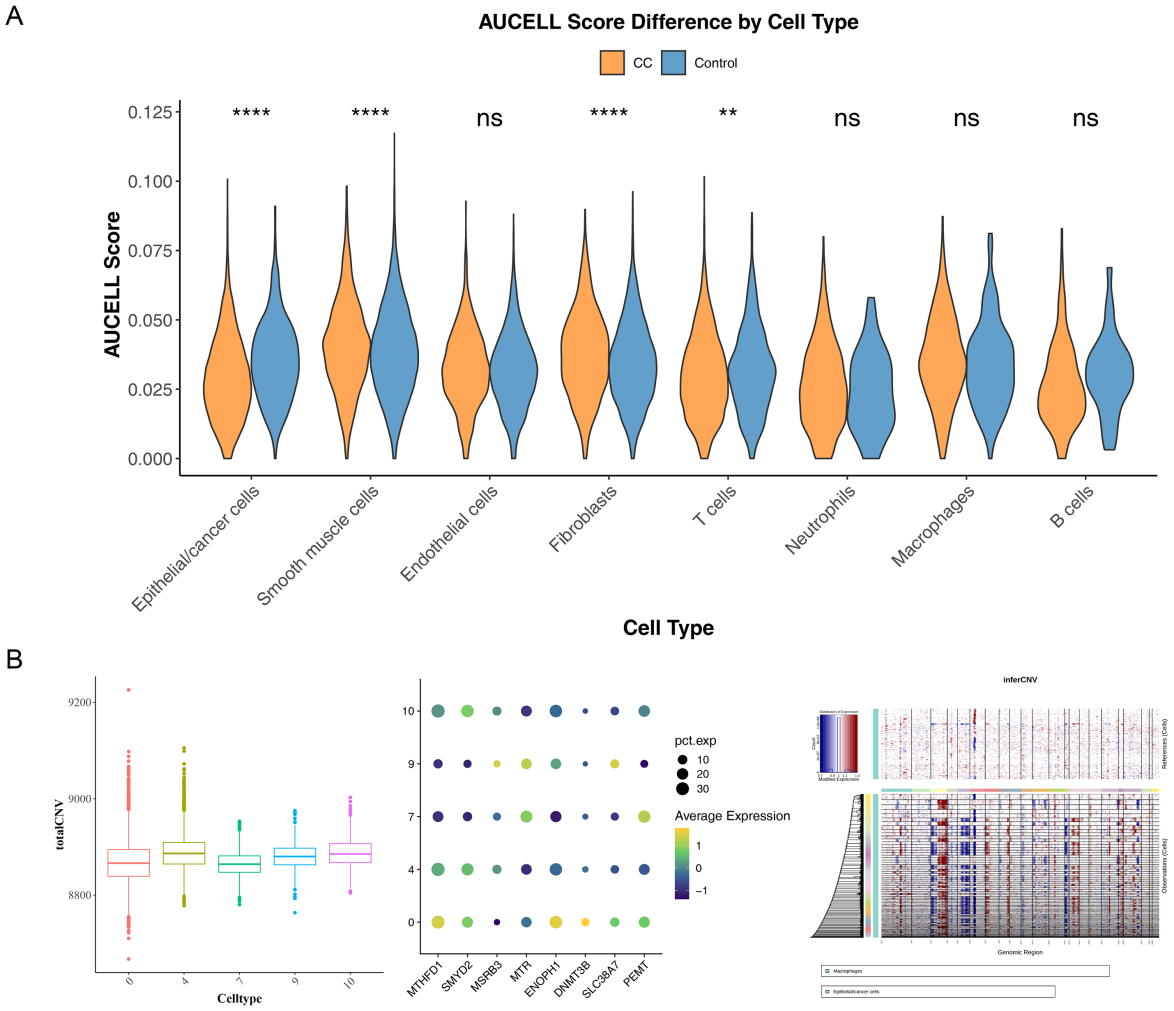
High-resolution validation of the prognostic MM-RG signature and cell malignancy. (A) Grouped violin plots comparing AUCell scores of the methionine metabolism pathway between CC and control groups across eight major cell types. Statistical significance was determined by the Wilcoxon rank-sum test (∗∗*p* < 0.01, ∗∗∗∗*p* < 0.0001, ns: not significant). (B) Integrated analysis of malignant sub-clusters. Left: Comparison of total CNV scores across epithelial sub-clusters (Clusters 0, 4, 7, 9, 10), identifying Clusters 4 and 10 as populations with high genomic instability. Middle: Dot plot illustrating the expression of the eight prognostic genes within these sub-clusters. Right: inferCNV heatmap displaying chromosomal copy number variations in epithelial clusters, with macrophages serving as the reference.

scRNA-seq analysis of the E-MTAB-11948 dataset identified eight distinct cell populations in the CC microenvironment (Fig. S2). The prognostic MM-RGs were found to be predominantly overexpressed in the epithelial/cancer cell population (Fig. S2). AUCell and inferCNV analyses further confirmed intensified MM within malignant epithelial sub-clusters (Clusters 4 and 10), which exhibited significantly higher expression of hub genes like MTHFD1 and SMYD2 compared to normal epithelial cells (Fig. 9). These high-resolution findings demonstrate that the prognostic signature is primarily driven by cell-intrinsic metabolic reprogramming within malignant cells.

## Discussion

Cervical cancer (CC) remains a major global health burden, especially in low- and middle-income regions, where access to screening and vaccination programs is limited. Despite advancements in surgical techniques, radiotherapy, and chemotherapy, the long-term prognosis of patients with advanced or recurrent CC remains suboptimal. This highlights the urgent need to identify novel prognostic markers and therapeutic targets. In this study, we comprehensively analyzed methionine metabolism-related genes (MM-RGs) in CC and constructed a robust eight-gene prognostic signature. The model was validated in an external cohort, where the high-risk group exhibited significantly poorer outcomes compared to the low-risk group (TCGA: HR = 2.365, *p* = 0.0063; GSE44001: HR = 6.176, *p* < 0.001), further supported by immune profiling, drug sensitivity prediction, and experimental validation.

### Biological and clinical relevance of the prognostic genes

The eight genes included in our model—MTHFD1, SMYD2, MSRB3, MTR, ENOPH1, DNMT3B, SLC38A7, and PEMT—are functionally diverse and participate in various facets of MM, epigenetic regulation, and cellular homeostasis.

MTHFD1 and MTR, both core components of the one-carbon metabolism pathway, contribute to tumorigenesis by supplying nucleotide precursors and modulating methylation status through regulation of S-adenosylmethionine (SAM) levels. The identified signature genes primarily function in metabolic and epigenetic regulation. Core components of the one-carbon cycle, such as MTHFD1 and MTR, provide essential nucleotide precursors and modulate methylation status *via* S-adenosylmethionine (SAM) regulation (Groth et al., 2016; Sullivan et al., 2021). Complementing this, methyltransferases SMYD2 and DNMT3B act at distinct epigenetic levels to promote transcriptional activation and DNA methylation patterns, respectively, collectively driving cervical carcinogenesis (Yi, Jiang & Fang, 2019; Jarrell et al., 2020; Duymich et al., 2016; Van den Boogaard et al., 2016; Zhang et al., 2024). Other components like ENOPH1 and MSRB3 support tumor resilience by optimizing methionine utilization and repairing oxidative damage, while transporters such as SLC38A7 and PEMT meet the biosynthetic and lipid metabolic demands of proliferating CC cells (Gale, Hehlmann & Hochhaus, Clara Bloomfield MD: Queen of AML; Agarwal et al., 2018; Long et al., 2017; Wu et al., 2006). In our study, PEMT was significantly upregulated in CC tissues, indicating that enhanced phospholipid methylation may support cancer cell proliferation by facilitating membrane synthesis and modulating lipid-derived signaling pathways.

Collectively, these genes highlight the multifaceted nature of MM in CC, extending beyond methylation to include redox control, amino acid transport, and lipid biosynthesis. Their coordinated upregulation suggests a broader metabolic adaptation that contributes to tumor survival and progression, reinforcing the relevance of MM as a promising target for future therapeutic strategies. Furthermore, the high-resolution evidence provided by scRNA-seq analysis reinforces the clinical relevance of our model. By demonstrating that the majority of prognostic MM-RGs are specifically enriched in malignant epithelial cells rather than the supportive stroma, this study confirms that the identified signature directly reflects the metabolic state of the tumor. The synchronization of high CNV levels with elevated MM-RG expression in specific malignant sub-clusters suggests a potential link between genomic instability and MM dysregulation in CC progression.

### Functional enrichment and immune microenvironment

Functional enrichment analysis revealed that CC is characterized by profound alterations in pathways associated with tumor invasion, metabolic reprogramming, and microenvironmental remodeling. Among the 39 significantly enriched pathways, several are directly implicated in tumor aggressiveness and immune dysregulation. In this section, we focus on the top five enriched pathways, beginning with Focal Adhesion and ECM–Receptor Interaction, which were highly enriched in the high-risk group (NES = 2.08 and 2.38, respectively; both p.adjust < 0.001) and are critical for cellular adhesion, motility, and tumor–stroma interactions.

Focal Adhesion is a central signaling hub that mediates the connection between cells and the extracellular matrix (ECM), primarily through integrins, focal adhesion kinase (FAK), paxillin, and other adaptor proteins. These complexes transduce mechanical and biochemical cues to orchestrate cell migration, proliferation, and survival by activating downstream cascades such as FAK/SRC and Rho GTPase signaling (Mishra & Manavathi, 2021). In the context of CC, aberrant activation of focal adhesion signaling has been closely linked with enhanced tumor cell invasion and metastasis. From a metabolic perspective, the dysregulation of MM provides an altered pool of S-adenosylmethionine (SAM), the universal methyl donor (Tassinari et al., 2024). This metabolic shift may lead to epigenetic remodeling, such as the promoter hypomethylation of genes encoding integrins and focal adhesion kinase (FAK), directly driving the epithelial–mesenchymal transition (EMT) and metastatic progression in high-risk CC patients (Zhang et al., 2022).

Oxidative phosphorylation (OXPHOS), a mitochondrial process responsible for ATP generation, has traditionally been considered downregulated in cancer due to the Warburg effect. However, emerging evidence suggests that many tumors, including CC, retain or even upregulate OXPHOS activity to meet bioenergetic and biosynthetic demands (Huang et al., 2019; Xie, Zhou & Chen, 2022). In this study, OXPHOS-related genes were significantly enriched in the high-risk group, implying increased mitochondrial respiration and metabolic flexibility. This metabolic adaptation is intrinsically linked to MM through several axes. First, the methionine cycle is coupled with the folate cycle to generate NADPH, which is essential for maintaining mitochondrial antioxidant capacity and supporting high OXPHOS activity (Orbe Jaramillo et al., 2020; Pranzini et al., 2021). Second, the assembly and functional integrity of electron transport chain complexes depend on SAM-mediated methylation of mitochondrial proteins (Rowland et al., 2025). Consequently, the intensified OXPHOS in high-risk patients may provide the bioenergetic resilience necessary to survive in hostile, nutrient-depleted microenvironments and resist chemotherapy. Such adaptations may support tumor growth under nutrient-limited or hypoxic conditions and have been associated with chemoresistance and immune suppression. For instance, enhanced OXPHOS activity can lead to elevated NAD^+^/NADH turnover and ROS production, which in turn stabilize HIF-1α and promote angiogenesis, a key feature of advanced CC (Rosenberger et al., 2022). Moreover, targeting OXPHOS with inhibitors such as IACS-010759 is being investigated in clinical trials and may represent a promising strategy in CC subtypes characterized by mitochondrial upregulation.

The ribosome pathway, reflecting increased ribosomal biogenesis and protein translation, was also enriched in the high-risk cohort. Cancer cells heavily depend on accelerated protein synthesis to sustain proliferation, adapt to stress, and modulate immune checkpoints (Wong et al., 2019). Ribosomal proteins and biogenesis factors (*e.g.*, RPL5, RPS6, NOP56) have been shown to act beyond their canonical roles, participating in oncogenic signaling, p53 regulation, and immune modulation (Kiyani et al., 2023). In CC, HPV oncoproteins can hijack the host translational machinery by promoting ribosome assembly and enhancing global protein output, thereby facilitating viral replication and tumorigenesis (Li, Ran & Qiao, 2023). Notably, ribosomal stress can paradoxically activate anti-tumor immune responses by increasing antigen presentation; however, in high-risk CC subtypes, this is often counteracted by immune checkpoint upregulation and T cell exhaustion.

Interestingly, the Parkinson’s disease pathway was also significantly enriched in the low-risk group (NES = −2.15, p.adjust < 0.001), which may reflect alterations in mitochondrial dynamics, oxidative stress response, and proteostasis—hallmarks shared between neurodegeneration and cancer (Zhang et al., 2019; Andreozzi, Ploner & Saral, 2020). Several genes commonly implicated in Parkinson’s disease, such as PINK1, PARK7 (DJ-1), and LRRK2, are also involved in tumor biology, particularly in regulating mitochondrial quality control, cell survival, and autophagy. In CC, oxidative stress induced by HPV infection and chronic inflammation may activate similar stress response pathways. For example, DJ-1 has been reported to exert antioxidative and anti-apoptotic functions in cancer cells under metabolic stress, thereby supporting tumor survival (Ofluoglu et al., 2017). Although the connection between neurodegenerative pathways and tumorigenesis is still under investigation, these findings underscore the shared metabolic vulnerabilities and potential for cross-disease therapeutic targets.

These pathways collectively form the “invasion-metabolism-immune escape” network in CC. Future therapies should target cross-nodal points (*e.g.*, FAK/ETC dual targeting + immune checkpoint blockade) to improve outcomes for advanced patients. Given growing evidence linking immune dysregulation to CC, understanding the immune landscape provides insights into its mechanisms (Schratz et al., 2023; Gautam et al., 2025).

Therefore, we utilized the CIBERSORT algorithm to analyze immune cell composition between high- and low-risk CC groups. Six immune cell types differed significantly between groups (*p* < 0.05). Among these, Regulatory T cells (Tregs) and Activated mast cells showed the strongest positive correlation with the risk score (*r* = −0.245, *p* < 0.05). Analysis of prognostic genes and differential immune cells showed the highest positive correlation with Memory B cells. Regulatory T cells (Tregs) play a crucial role in the development and progression of cervical cancer.

Studies demonstrate that the frequency of Tregs in peripheral blood is significantly higher in cervical cancer patients (11.00 ± 19.79%) compared to healthy individuals (1.71 ± 8.91%), with a further increase observed in high-risk HPV (HR-HPV) positive patients (10.90 ± 15.97% *vs.* 1.72 ± 9.19%) (Chetty-Sebastian & Assounga, 2023). By suppressing the host’s specific immune response against HPV, Tregs promote persistent viral infection and progression to cervical cancer. Further analysis revealed a significant elevation in the proportion of CD4^+^CD25^+^ T cells in the peripheral blood of patients with cervical cancer and precancerous lesions (Visser et al., 2007). Moreover, in mouse models of cervical cancer, tumors exhibiting non-response to anti-PD-L1 therapy showed heightened immunosuppressive activity, characterized by significantly elevated expression of Treg-related molecules such as Foxp3. This suggests Tregs may be a key factor influencing immunotherapy efficacy (Xu et al., 2021). The correlation between MM and immune suppression can be explained by ‘nutrient competition’ and metabolic signaling. CC cells exhibit a ‘methionine addiction,’ rapidly consuming extracellular methionine and SAM. This depletion can impair the methylation patterns and functional maturation of effector T cells while paradoxically promoting the differentiation of immunosuppressive Tregs (Sharma et al., 2025). Furthermore, methionine metabolites like homocysteine may act as signaling molecules that trigger immunosuppressive pathways in mast cells, collectively fostering an environment conducive to immune escape and rapid tumor progression (Bian et al., 2020).

This study indicates (in our research) that regulatory T cells exhibit the strongest positive correlation with mast cells. Mast cells promote tumor angiogenesis and lymphangiogenesis by releasing pro-angiogenic factors such as vascular endothelial growth factor (VEGF) and VEGF-C (Utrera-Barillas et al., 2010). We therefore speculate that the synergistic interaction between Tregs and mast cells may be a significant reason for the non-response to anti-PD-L1 therapy.

In summary, the enrichment of pathways related to energy metabolism, translation, and oxidative stress response in high-risk CC patients reflects a complex adaptive landscape that supports tumor proliferation, immune escape, and therapy resistance. These findings not only validate the prognostic relevance of MM-RGs but also suggest potential targets for metabolic and immunotherapeutic intervention.

### Therapeutic implications

Drug sensitivity analysis suggested Cediranib, BI-2536, and Tozasertib as potential candidate agents based on in-silico predictions. Among the identified therapeutic agents, Cediranib, a potent VEGFR inhibitor, showed significant efficacy in the high-risk group, with a 34.4% and 31.0% reduction in predicted IC50 values for Cediranib and BI-2536, respectively, compared to the low-risk group (both *p* < 0.001). The potential clinical relevance of Cediranib is hypothesized to involve a synergistic interplay with MM. As methionine is an essential amino acid with tumor cells displaying a marked ’methionine addiction,’ Cediranib-mediated anti-angiogenesis could further restrict the nutrient supply (Batchelor et al., 2010) within the tumor microenvironment, selectively starving high-risk cells that are heavily dependent on this metabolic axis (Friese-Hamim et al., 2024). Furthermore, the hypoxia induced by vascular inhibition typically exacerbates oxidative stress; since MM-RGs such as MSRB3 and enzymes involved in glutathione synthesis are critical components of the antioxidant defense system, the combination of MM dysregulation and Cediranib treatment is speculated to potentially induce synthetic lethality in CC cells, although direct experimental evidence is currently lacking. In addition to Cediranib, BI-2536 and Tozasertib emerged as high-potential therapeutic candidates for the high-risk group, with their efficacy likely rooted in the intricate crosstalk between the cell cycle and MM. BI-2536, by inhibiting PLK1, may suppress the activity of MAT2A (Gounder et al., 2025), the rate-limiting enzyme in the methionine cycle, thereby depleting the S-adenosylmethionine (SAM) pool and inducing methylation stress in metabolically active CC cells (Jiang et al., 2025). Similarly, Tozasertib-mediated inhibition of Aurora kinases is predicted to interfere with the MYC-driven one-carbon metabolic flux (Dong et al., 2020) and impairs the transsulfuration pathway, which is essential for glutathione-mediated antioxidant defense (Krieg et al., 2024). These biochemical links suggest that targeting mitotic regulators could exploit the metabolic vulnerabilities of high-risk tumor cells, providing a strong rationale for personalized treatment strategies based on MM signatures.

Importantly, the identification of MM-RGs as modulators of both tumor cell-intrinsic and immune-related processes opens new avenues for combined metabolic-immunotherapeutic strategies. For instance, inhibiting one-carbon metabolism may sensitize tumors to immunotherapy by reversing immune evasion and restoring T cell function. It is important to note that these proposed mechanisms are based on integrated bioinformatic analysis and computational modeling. Further molecular biology experiments are essential to validate whether these agents directly modulate methionine metabolic flux in cervical cancer cells.

### Experimental validation and biological credibility

qRT-PCR analysis of clinical samples confirmed the overexpression of MTHFD1, MTR, MSRB3, and ENOPH1 in tumor tissues, consistent with transcriptomic data. Although SMYD2 did not reach statistical significance, its upward trend supports its inclusion in the prognostic model. These preliminary results provide small-scale support for the expression trends predicted by the model and highlight the translational potential of MM-RGs as biomarkers.

### Research significance and limitations

The prognostic model developed in this study, based on MM-RGs, holds significant potential for clinical translation. By integrating transcriptomic data with clinical outcomes and experimental validation, we constructed a risk stratification framework capable of accurately predicting disease-free survival in CC patients. This model not only reflects metabolic heterogeneity but also captures key tumor biological features such as epigenetic dysregulation, immune evasion, and microenvironment remodeling. Importantly, high-risk patients identified by the model exhibited distinct immunological profiles and pathway enrichments, suggesting that the model may also serve as a predictive tool for therapy responsiveness, particularly in the context of metabolic and immuno-oncology strategies. The generalizability of the risk signature was rigorously tested through cross-platform validation using the GSE44001 microarray dataset. While further validation in localized cohorts would be ideal, a systematic review of public repositories indicated that most CC transcriptomic datasets lack sufficient survival metadata or consistent clinical annotations. The high degree of consistency between the TCGA (RNA-seq) and GSE44001 (microarray) results confirms that the eight-gene model is not overfitting to a specific platform and possesses strong potential for clinical translation.

While our study presents a comprehensive analysis of MM in CC, several limitations should be acknowledged:

Retrospective design: The model is based on retrospective data from public databases. Furthermore, the sample sizes for our specific validation components present certain limitations. The single-cell RNA-seq analysis was conducted using a single dataset (E-MTAB-11948), which may not fully represent the vast heterogeneity of the CC microenvironment across different populations. Additionally, our clinical validation *via* qRT-PCR was restricted to a small cohort of five paired samples, which limits the statistical power and the ability to generalize these expression trends to all clinical stages. Prospective validation in independent clinical cohorts is necessary. Lack of *in vivo* validation: Although we confirmed gene expression by qRT-PCR, functional assays in cell lines or animal models are needed to elucidate mechanistic roles. Additionally, our identification of potential therapeutic agents is based entirely on in-silico drug sensitivity predictions. This computational approach lacks direct experimental validation through *in vitro* or *in vivo* assays; therefore, the predicted clinical efficacy of candidates like Cediranib and BI-2536 must be interpreted with caution until confirmed by further experimental evidence. Ethnic bias: The TCGA and GEO datasets primarily contain samples from specific populations, which may limit the generalizability of the findings. Treatment heterogeneity: Clinical details regarding treatment regimens were incomplete, precluding stratified analysis based on therapy.

Despite these limitations, our study provides a solid foundation for future research into MM and its role in CC progression and therapy.

To facilitate the translational application of these computational findings, a structured experimental roadmap is proposed for the validation of high-potential candidates such as Cediranib. Future studies should prioritize comparing the sensitivity of Cediranib in CC cell lines with divergent risk profiles (*e.g.*, those with high *vs.* low expression of the eight-gene signature). Initial validation would involve verifying the expression of key MM-RGs, such as MTHFD1 and MTR, *via* qRT-PCR or Western Blot to simulate the risk model. Subsequently, cell viability and colony formation assays can be employed to determine the half-maximal inhibitory concentration (IC50) across these cell lines. It is hypothesized that high-risk cell lines will exhibit significantly greater sensitivity to Cediranib. Moreover, investigating post-treatment changes in intracellular SAM/SAH ratios, global DNA methylation levels, and reactive oxygen species (ROS) production would clarify whether the cytotoxic effects of these agents are indeed mediated through the modulation of methionine-related pathways. Such experimental evidence will be crucial for bridging the gap between bioinformatic prediction and clinical implementation.

## Conclusions

This study established a novel, methionine metabolism-based prognostic model for CC and highlighted the intricate interplay between MM, immune modulation, and therapeutic response. By integrating transcriptomic data and clinical validation, we developed a robust eight-gene signature that effectively stratified patients into risk groups with significant differences in survival outcomes.

Our findings provide novel insights into the metabolic-immune landscape of CC, revealing that the prognostic signature is primarily driven by cell-intrinsic metabolic reprogramming within malignant epithelial cells. Overall, our study provides novel insights into the metabolic-immune landscape of cervical cancer and establishes a gene signature that has potential clinical applicability pending further validation in personalized medicine. Future prospective studies and mechanistic experiments are warranted to further validate the practical utility of the model and to explore methionine metabolism as a therapeutic target.

##  Supplemental Information

10.7717/peerj.21538/supp-1 Supplemental Information 1 Code

10.7717/peerj.21538/supp-2 Supplemental Information 2 Pathology Report Certificate

10.7717/peerj.21538/supp-3 Supplemental Information 3 List of 68 methionine metabolism-related genes (MM-RGs) utilized in this study This gene set was compiled from the Molecular Signatures Database (MSigDB) and Gene Ontology (GO) annotations. These genes served as the background set for intersection with differentially expressed genes (DEGs) to identify biologically relevant candidate prognostic markers.

10.7717/peerj.21538/supp-4 Supplemental Information 4 Detailed clinical and histopathological characteristics of the five cervical cancer patients (n=5 paired samples) used for qRT-PCR validation This table provides comprehensive background information for the clinical samples, including patient age, histological category (squamous cell carcinoma or adenocarcinoma), FIGO stage (IB1-IIA1), tumor grade (G2-G3), and corresponding pathology identifiers. These samples were utilized to validate the expression trends of key methionine metabolism-related genes (MM-RGs) via qRT-PCR.

10.7717/peerj.21538/supp-5 Supplemental Information 5 Primer sequences and parameters used for qRT-PCR validation This table lists the detailed information of the primers used to validate the expression levels of key methionine metabolism-related genes (MM-RGs) and the internal reference gene (GAPDH), including gene accession numbers, primer sequences, amplicon lengths, primer directions, and melting temperatures (Tm).

10.7717/peerj.21538/supp-6 Supplemental Information 6 Detailed results of Gene Ontology (GO) enrichment analysis for the 34 candidate methionine metabolism-related genes (MM-RGs) This table provides a comprehensive list of enrichment parameters, including ONTOLOGY (BP: Biological Process; CC: Cellular Component; MF: Molecular Function), Term ID and Description, Background Ratio (BgRatio), p-value, Benjamini-Hochberg adjusted p-value (p.adjust/FDR), q-value, specific gene symbols (geneID), gene count, and rich factor. Enrichment terms were considered statistically significant based on a threshold of FDR < 0.05.

10.7717/peerj.21538/supp-7 Supplemental Information 7 List of 57 candidate chemotherapeutic agents with significantly differential in-silico sensitivity between high-risk and low-risk groups This table summarizes the results of the oncoPredict analysis, identifying 57 drugs with significantly different predicted IC50 values (*p* < 0.05) between high-risk and low-risk CC patients. The table includes drug names (or database identifiers) and their corresponding p-values. These agents are suggested as potential therapeutic options for high-risk individuals based on computational predictions.

10.7717/peerj.21538/supp-8 Supplemental Information 8 Histogram of FDR distribution for all detected genes The plot illustrates the statistical distribution of significance levels across the whole transcriptome in the TCGA-CESC cohort. The x-axis represents the −log _ 10 transformed FDR values, and the y-axis represents the gene count. The red dashed vertical line indicates the conventional significance threshold of FDR = 0.05 (−log _ 10 FDR≈1.30). The distribution spans a broad range from 0 to 124.54, with an interquartile range (IQR) of 0.26 to 4.63. A substantial proportion of genes are distributed to the right of the threshold with a pronounced tail, indicating extensive and highly significant transcriptomic reprogramming in CC, which supports the statistical validity of the 11,955 identified DEGs.

10.7717/peerj.21538/supp-9 Supplemental Information 9 Technical workflow and cell type annotation of the scRNA-seq dataset (A) Violin plots illustrating quality control metrics (nFeature_RNA, nCount_RNA, and percent.mt) for the E-MTAB-11948 dataset before and after data filtering. (B) PCA dimensionality reduction scatter plot, with individual cells color-coded by their original sample identity. (C) UMAP visualization of 51,324 high-quality cells annotated into eight distinct cell populations based on canonical markers. (D) Dot plot showing the cell-type-specific expression distribution of the eight prognostic MM-RGs, highlighting their enrichment in the epithelial/cancer cell population.

10.7717/peerj.21538/supp-10 Supplemental Information 10 Original data of the cell experiments

## Abbreviations

CC: Cervical Cancer
EOCC: Early-Onset Cervical Cancer
LOCC: Late-Onset Cervical Cancer
HPV: Human Papillomavirus
TME: Tumor Microenvironment
CAF: Cancer-Associated Fibroblast
TILs: Tumor-Infiltrating Lymphocytes
Tregs: Regulatory T Cells
NK Cells: Natural Killer Cells
DCs: Dendritic Cells
scRNA-seq: Single-Cell RNA Sequencing
DEGs: Differentially Expressed Genes
GO: Gene Ontology
KEGG: Kyoto Encyclopedia of Genes and Genomes
GSEA: Gene Set Enrichment Analysis
GSVA: Gene Set Variation Analysis
PCA: Principal Component Analysis
UMAP: Uniform Manifold Approximation and Projection
CNV: Copy Number Variation
SNP: Single Nucleotide Polymorphism
FDR: False Discovery Rate
TPM: Transcripts Per Million
FPKM: Fragments Per Kilobase of transcript per Million mapped reads
H&E: Hematoxylin and Eosin
IHC: Immunohistochemistry
qPCR: Quantitative Polymerase Chain Reaction
IF: Immunofluorescence
EMT: Epithelial–Mesenchymal Transition
PD-L1: Programmed Death-Ligand 1
CTLA-4: Cytotoxic T-Lymphocyte-Associated Protein 4
MSI: Microsatellite Instability
TMB: Tumor Mutation Burden
ICIs: Immune Checkpoint Inhibitors
MAT2A: Methionine Adenosyltransferase 2A
DNMT3B: DNA (Cytosine-5)-Methyltransferase 3 Beta
SMYD2: SET and MYND Domain Containing 2
HDAC: Histone Deacetylase
lncRNA: Long Non-Coding RNA
HOTAIR: HOX Transcript Antisense RNA
DESeq2: Differential Expression Sequencing 2 (R package)
oncoPredict: R package for drug sensitivity prediction
clusterProfiler: R/Bioconductor Package for Functional Enrichment
GEO: Gene Expression Omnibus
TCGA: The Cancer Genome Atlas
ROC: Receiver Operating Characteristic
AUC: Area Under the Curve
OS: Overall Survival
PFS: Progression-Free Survival
HR: Hazard Ratio
CI: Confidence Interval
MM: Methionine Metabolism
MM-RGs: Methionine Metabolism-related genes

## Additional Information and Declarations

### Data Availability

The following information was supplied regarding data availability: The raw data are available in the Supplemental Files. The data that was used in this study is available at: - TCGA-CESC: https://portal.gdc.cancer.gov/projects/TCGA-CESC
- GTEx: https://gtexportal.org/home/datasets
- GEO: GSE44001
