# Supplementary material for: A prognostic signature based on methionine metabolism-related genes for cervical cancer: integrated transcriptomic and experimental validation
Source: PeerJ. 2026 Jul 21;14:e21538. doi: 10.7717/peerj.21538 (PMC13398394; doi:10.7717/peerj.21538)
Supplement: Supplemental Information 2 [file peerj-14-21538-s002.pdf]

Pathology Report Certificate for Patient A
(Pathology Number: B2500832)

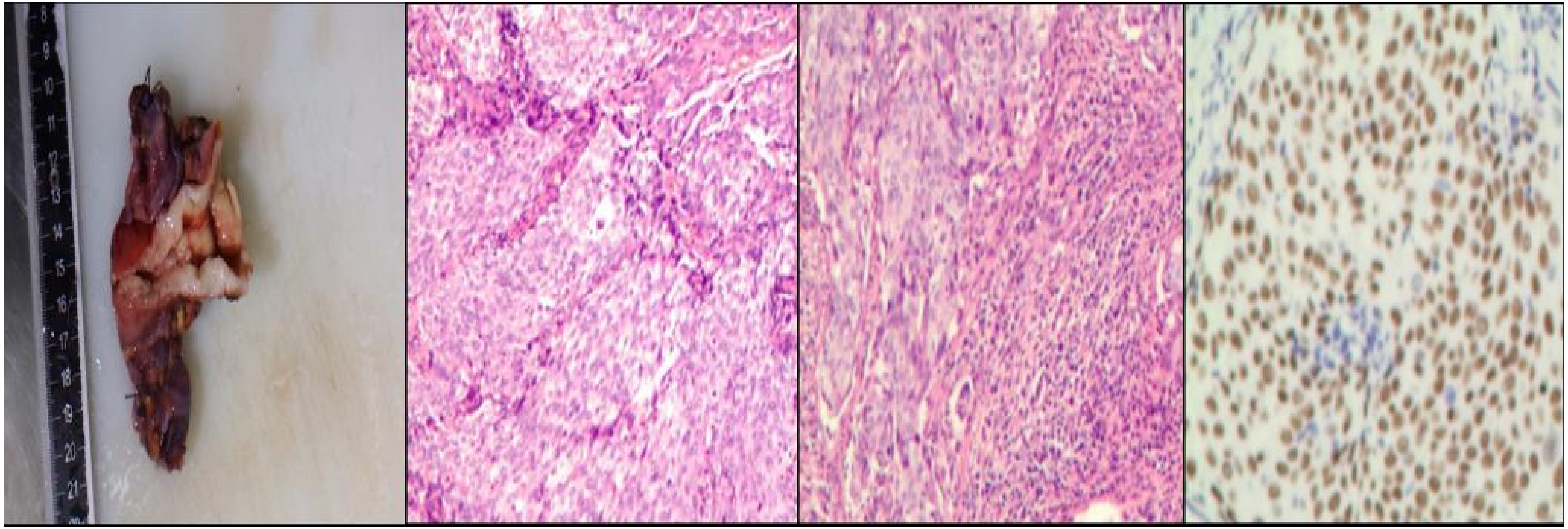

TPathological Diagnosis((Specimen from Total Hysterectomy with Bilateral Adnexectomy)Patient A | Pathology Number: B2500832**)** **:** umor Site: Cervix uteri; Tumor Size and Type: 17 × 14 × 6 mm; ulcerative type, Histological Type: HPV-associated squamous cell carcinoma; Tumor Grade: Poorly differentiated Depth of Invasion: More than two-thirds of the cervical stromal wall, approximately 1 mm from the external cervical serosa; Tumor Involvement: Involvement of the lower uterine segment and endometrium;no carcinoma involvement in either adnexa; Lymphovascular Space Invasion (LVSI): Present; Perineural Invasion: Not observed; Surgical Margins: No residual carcinoma at vaginal cuff margins or bilateral parametrial margins; Adnexa: Bilateral fallopian tubes and ovaries show atrophic changes; Lymph Node Examination:Parametrial lymph node: 1 node examined, no metastasis (0/1) Left pelvic lymph nodes: 5 nodes examined, no metastasis (0/5); Right pelvic lymph nodes: 11 nodes examined, no metastasis (0/11) Immunohistochemistry Results (Block No. B2500832-7):CD31: Positive (vascular); D2-40: Positive (vascular); Ki-67: Positive (~60%); P40: Positive; P16: Positive; P63: Positive; S-100: Positive (nerve structures); P53: Positive (wild-type expression pattern).

Reviewed by: Dr. Huang Tingyu

Pathology Report Certificate for Patient B
（Pathology Number: B2501055）

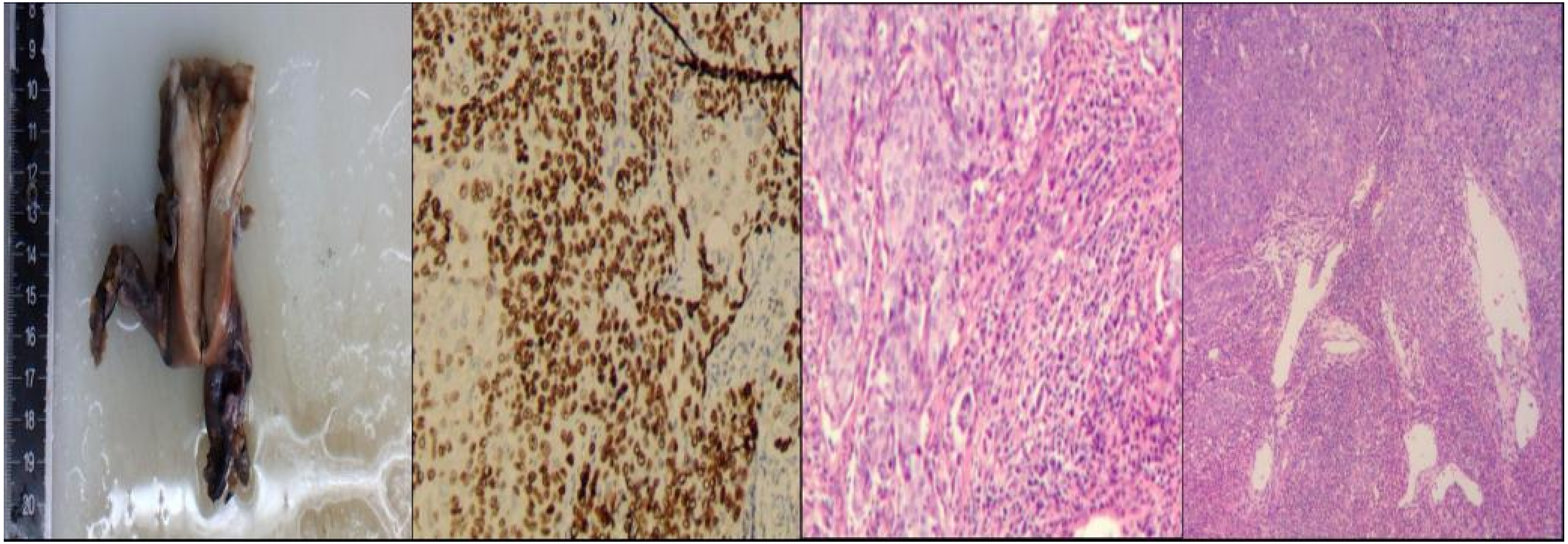

Pathological Diagnosis(Specimen from Radical Hysterectomy with Bilateral Adnexectomy)：Specimen Type: Surgical excision specimen；Tumor Location: Cervix；Tumor Size: 20 × 17 × 12 mm；Gross Morphology: Exophytic, cauliflower-like mass；Histological Type: HPV-associated squamous cell carcinoma；Tumor Grade: Moderately to poorly differentiated；Depth of Invasion: Invasion into the inner one-third of the cervical canal wall；Tumor Extent: No carcinoma involvement in the lower uterine segment, vaginal fornix, or bilateral adnexa；Lymphovascular Space Invasion (LVSI): Not identified；Perineural Invasion: No definite perineural invasion observed；Surgical Margins: No residual tumor at the vaginal resection margin or bilateral parametrium；Uterine Body: Atrophic endometrium, no specific pathological changes in the myometrium；Adnexa: Bilateral fallopian tubes: Stromal vascular dilation and congestion，Bilateral ovaries: Corpus albicans and inclusion cyst formation；Immunohistochemistry Results (Block No. B2501055-4):CD34: Positive (vascular)，Ki-67: Positive (~70%)，P40: Positive，P63: Positive，S-100: Positive (nerve structures)，P16: Positive.

Reviewed by: Dr. Huang Tingyu

Pathology Report Certificate for Patient C
(Pathology Number: B2500963)

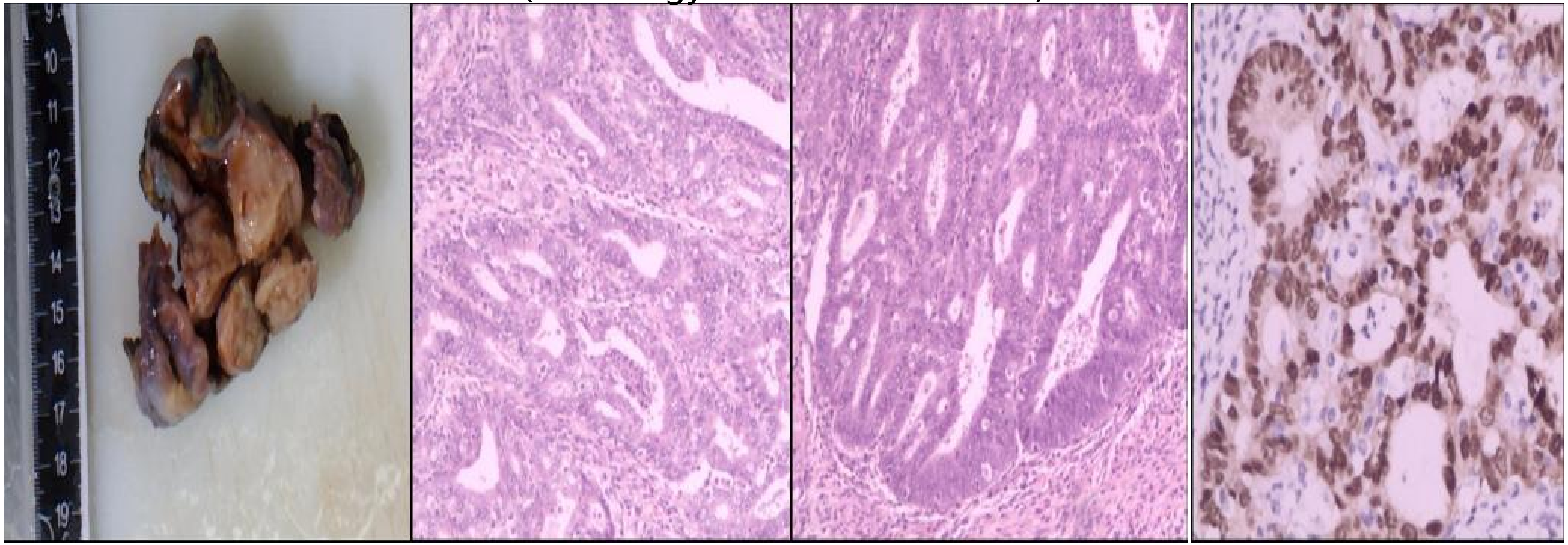

Pathological Diagnosis(Specimen from Radical Hysterectomy with Bilateral Adnexectomy):Tumor Site: Cervix;Gross Morphology: Endophytic infiltrative type;Tumor Dimensions: 20 mm × 10 mm × 20 mm;Histological Type: Based on immunohistochemistry and special stains, findings support a diagnosis of cervical adenocarcinoma, likely HPV-associated;Depth of Invasion: Full-thickness invasion of cervical wall, 5 mm from the endocervical surface;Tumor Extent: No carcinoma involvement in the lower uterine segment;Lymphovascular Space Invasion (LVSI): Present;Perineural Invasion: Present;Surgical Margins: Small amount of tumor observed at vaginal margin,No residual carcinoma at bilateral parametrial margins;Other Findings: Uterus: Leiomyoma; atrophic endometrium;Bilateral fallopian tubes: Chronic salpingitis;Bilateralovaries: Corpus albicans formation;Immunohistochemistry Results (Block No. B2500963-7):CA9: Partially positive,S-100: Positive (nerve tissue),PAX-8: Positive,CD34: Positive (vascular structures),CEA: Focally positive,ER (Estrogen Receptor): Negative,Ki-67: Positive (approx. 30–40%),MUC6: Negative,P16: Positive (diffuse staining),PR (Progesterone Receptor): Negative,P53: Positive (mutant-type staining pattern),MUC5AC: Partially positive.

Reviewed by: Dr. Huang Tingyu

Pathology Report Certificate for Patient D
(Pathology Number:B2501162)

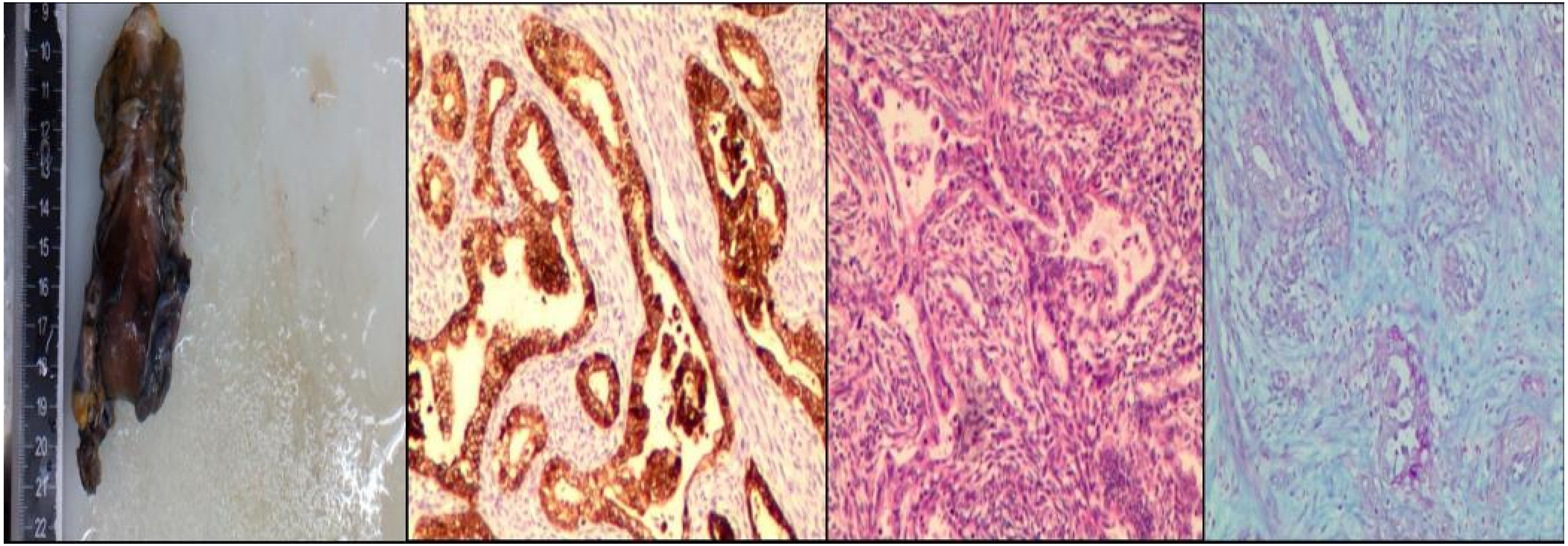

Pathological Diagnosis(Specimen from Radical Hysterectomy with Right Adnexectomy):Tumor Site: Cervix;Gross Morphology: Endophytic infiltrative type;Tumor Dimensions: 35 mm × 30 mm × 20 mm;Histological Type: Based on immunohistochemistry and special staining, findings support a diagnosis of cervical adenocarcinoma, likely HPV-associated.(Note: Correlation with clinical findings is recommended. If necessary, perform histological HPV-related testing for further confirmation.);Depth of Invasion: Full-thickness invasion of the cervical wall; the distance from the endocervical lining is approximately 13 mm;Tumor Extent: Involvement of the lower uterine segment and vaginal fornin,No evidence of carcinoma in the right adnexa;Lymphovascular Space Invasion (LVSI): Present Perineural Invasion: Present;Surgical Margins: Small amount of tumor detected at the vaginal resection margin,No residual carcinoma seen at bilateral parametrial margins;Other Findings: Uterus: Leiomyoma; atrophic endometrium,Right fallopian tube: Chronic salpingitis,Right ovary: Corpus albicans formation Immunohistochemistry Results(Block No.: B2501162-7):CA9: Partially positive,S-100: Positive (nerve tissue),PAX-8: Positive,CD34: Positive (vascular structures),CEA: Focally positive,ER (Estrogen Receptor): Negative,Ki-67: Positive (approx. 30–40%),MUC6: Negative,P16: Positive (diffuse staining),PR (Progesterone Receptor): Negative,P53: Positive (mutant-type staining pattern),MUC5AC: Partially positive.Special Staining Results(Block No.: B2501162-7):AB/PAS: Focally positive.

Reviewed by: Dr. Huang Tingyu

Pathology Report Certificate for Patient E
(Pathology Number: B2501125)

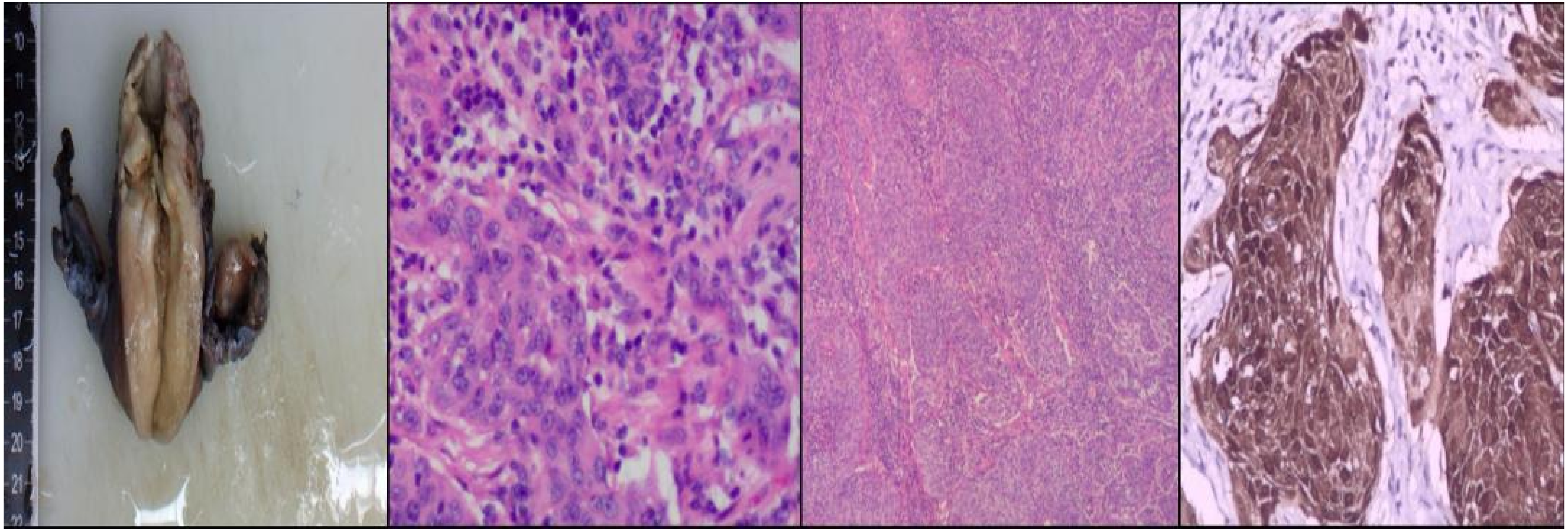

Pathological Diagnosis(Specimen from Radical Hysterectomy with Bilateral Adnexectomy):Tumor Size: 32 × 25 × 20 mm,Gross Type: Endophytic infiltrative type,Histological Type: HPV-associated squamous cell carcinoma,Tumor Grade: Poorly to moderately differentiated,Depth of Invasion: Full-thickness invasion of the cervical canal wall,Tumor Involvement: Tumor extension to the lower uterine segment and vaginal fornix,No carcinoma involvement in either adnexa;Lymphovascular Space Invasion (LVSI): Present;Perineural Invasion: Present;Surgical Margins: Vaginal margin: Areas of severe squamous intraepithelial dysplasia observed,Left parametrial margin: Carcinoma detected,Right parametrial margin: No residual carcinoma,Concurrent Lesions: Cervix shows chronic inflammatory changes with squamous metaplasia of glandular epithelium and retention cysts;Uterine Body:Proliferative endometrium,No specific pathological changes identified in the myometrium;Adnexa (Bilateral):Fallopian Tubes: Stromal vascular dilation and congestion;Ovaries: Corpus albicans and inclusion cyst formation

Immunohistochemistry Results(Block No.: B2501125-6):CD34: Positive (vascular structures),P40: Positive,Ki-67: Positive (~90%),P16: Positive (diffuse staining),P63: Positive,S-100: Positive (nerve structures).

Reviewed by: Dr. Huang Tingyu
