# Supplementary material for: A prognostic signature based on methionine metabolism-related genes for cervical cancer: integrated transcriptomic and experimental validation
Source: PeerJ. 2026 Jul 21;14:e21538. doi: 10.7717/peerj.21538 (PMC13398394; doi:10.7717/peerj.21538)
Supplement: Supplemental Information 5 — This table lists the detailed information of the primers used to validate the expression levels of key methionine metabolism-related genes (MM-RGs) and the internal reference gene (GAPDH), including gene accession numbers, primer sequences, amplicon lengths, primer directions, and melting temperatures (Tm). [file peerj-14-21538-s005.docx]

| **Gene number** | **Gene name** | **Primer sequence** | **Product length（bp）** | **Primer direction** | **Melting temperature（Tm, °C）** |
| --- | --- | --- | --- | --- | --- |
| [NM_005956.4](https://www.ncbi.nlm.nih.gov/entrez/viewer.fcgi?db=nucleotide&id=1418483987) | MTHFD1 F | CCCGAAGCTCAACACAAAGC | 265 | 5‘→3’ | 60.04 |
| [NM_005956.4](https://www.ncbi.nlm.nih.gov/entrez/viewer.fcgi?db=nucleotide&id=1418483987) | MTHFD1 R | CCTGTTCTGTTTCAGGGTCCA | 265 | 5‘→3’ | 59.86 |
| [XM_054337757.1](https://www.ncbi.nlm.nih.gov/entrez/viewer.fcgi?db=nucleotide&id=2462511584) | SMYD2 F | CCACCAAGGACAAGTAAGCAC | 94 | 5‘→3’ | 59.12 |
| [XM_054337757.1](https://www.ncbi.nlm.nih.gov/entrez/viewer.fcgi?db=nucleotide&id=2462511584) | SMYD2 R | TGTGTTCCAGGCCCATGTAG | 94 | 5‘→3’ | 59.67 |
| [XM_054371653.1](https://www.ncbi.nlm.nih.gov/entrez/viewer.fcgi?db=nucleotide&id=2462531123) | MSRB3 F | TGCCACGTTAGGGTGTGACC | 212 | 5‘→3’ | 62.39 |
| [XM_054371653.1](https://www.ncbi.nlm.nih.gov/entrez/viewer.fcgi?db=nucleotide&id=2462531123) | MSRB3 R | TAAGGTGTCAGTGAGGCTGC | 212 | 5‘→3’ | 59.68 |
| [NM_000254.3](https://www.ncbi.nlm.nih.gov/entrez/viewer.fcgi?db=nucleotide&id=1844139169) | MTR F | TATGGTGCTGGATGGAGGGA | 262 | 5‘→3’ | 60.03 |
| [NM_000254.3](https://www.ncbi.nlm.nih.gov/entrez/viewer.fcgi?db=nucleotide&id=1844139169) | MTR R | CATCCGGTAGGCCAAGTGTT | 262 | 5‘→3’ | 60.04 |
| [NM_021204.5](https://www.ncbi.nlm.nih.gov/entrez/viewer.fcgi?db=nucleotide&id=1519246165) | ENOPH1 F | TCCAGGTGTGCAGAAGTGTC | 254 | 5‘→3’ | 59.89 |
| [NM_021204.5](https://www.ncbi.nlm.nih.gov/entrez/viewer.fcgi?db=nucleotide&id=1519246165) | ENOPH1 R | GAAAGCAATCGGGGTTGTGG | 254 | 5‘→3’ | 59.76 |
| [NM_001256799.3](https://www.ncbi.nlm.nih.gov/entrez/viewer.fcgi?db=nucleotide&id=1676318038) | internal reference H-GAPDH F | ATGGGCAGCCGTTAGGAAAG | 135 | 5‘→3’ | 60.39 |
| [NM_001256799.3](https://www.ncbi.nlm.nih.gov/entrez/viewer.fcgi?db=nucleotide&id=1676318038) | internal reference H-GAPDH R | AGGAAAAGCATCACCCGGAG | 135 | 5‘→3’ | 60.04 |
