# Supplementary figures and images for: A prognostic signature based on methionine metabolism-related genes for cervical cancer: integrated transcriptomic and experimental validation
Source: PeerJ. 2026 Jul 21;14:e21538. doi: 10.7717/peerj.21538 (PMC13398394; doi:10.7717/peerj.21538)

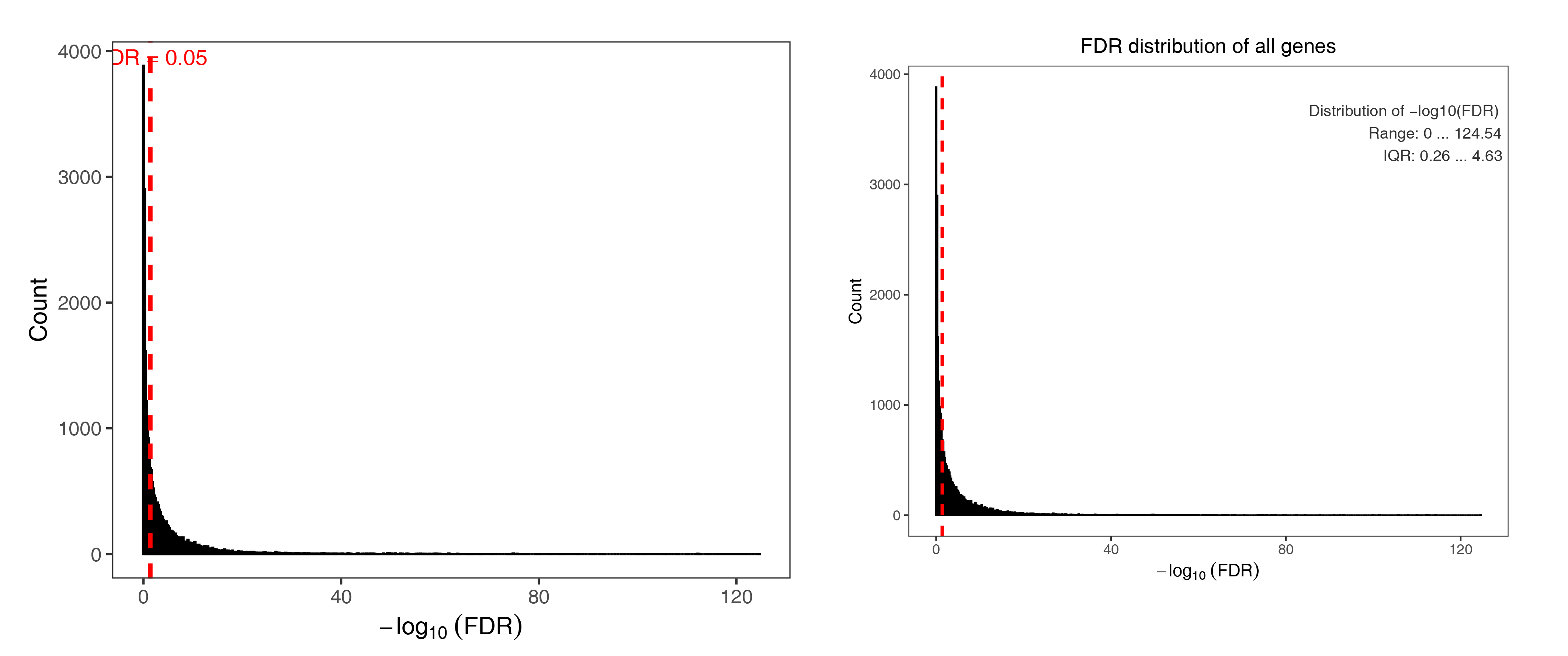

Supplement: Supplemental Information 8 — The plot illustrates the statistical distribution of significance levels across the whole transcriptome in the TCGA-CESC cohort. The x-axis represents the −log _ 10 transformed FDR values, and the y-axis represents the gene count. The red dashed vertical line indicates the conventional significance threshold of FDR = 0.05 (−log _ 10 FDR≈1.30). The distribution spans a broad range from 0 to 124.54, with an interquartile range (IQR) of 0.26 to 4.63. A substantial proportion of genes are distributed to the right of the threshold with a pronounced tail, indicating extensive and highly significant transcriptomic reprogramming in CC, which supports the statistical validity of the 11,955 identified DEGs. [file peerj-14-21538-s008.png]

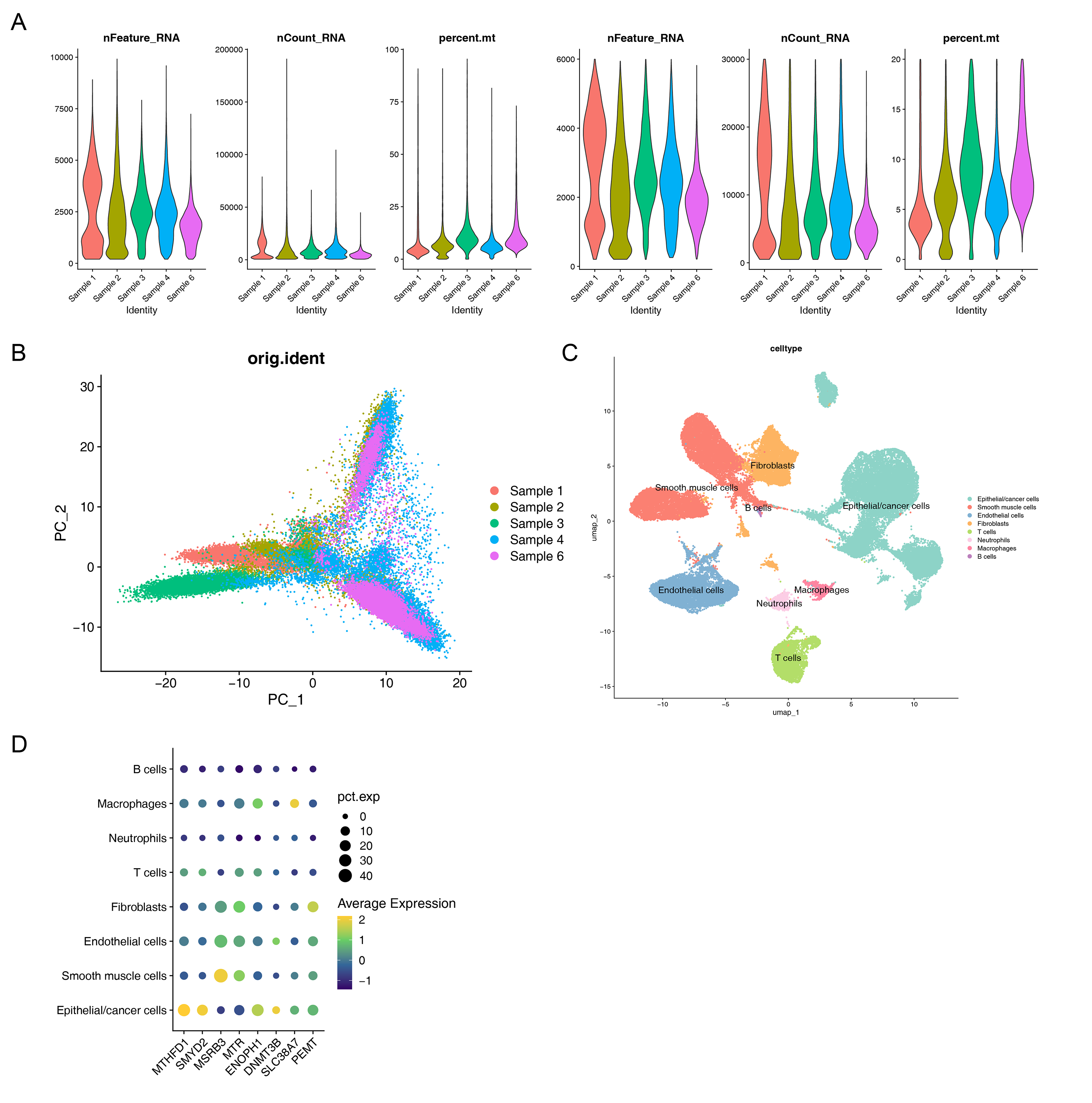

Supplement: Supplemental Information 9 — (A) Violin plots illustrating quality control metrics (nFeature_RNA, nCount_RNA, and percent.mt) for the E-MTAB-11948 dataset before and after data filtering. (B) PCA dimensionality reduction scatter plot, with individual cells color-coded by their original sample identity. (C) UMAP visualization of 51,324 high-quality cells annotated into eight distinct cell populations based on canonical markers. (D) Dot plot showing the cell-type-specific expression distribution of the eight prognostic MM-RGs, highlighting their enrichment in the epithelial/cancer cell population. [file peerj-14-21538-s009.png]
